# Selective and Controllable Cracking of Polyethylene Waste by Beta Zeolites with Different Mesoporosity and Crystallinity

**DOI:** 10.1002/advs.202404426

**Published:** 2024-07-08

**Authors:** Yanchao Liu, Weijiong Dai, Jiajun Zheng, Yanze Du, Quanhua Wang, Niklas Hedin, Bo Qin, Ruifeng Li

**Affiliations:** ^1^ Research Centre of Energy Chemical & Catalytic Technology Taiyuan University of Technology Taiyuan 030024 China; ^2^ SINOPEC Dalian Research Institute of Petroleum & Petrochemicals Co., Ltd Dalian 116045 China; ^3^ Department of Materials and Environmental Chemistry Stockholm University Stockholm SE‐10691 Sweden

**Keywords:** beta zeolite, bulky molecules, embryonic, hydrocracking, plastic waste

## Abstract

Waste plastics bring about increasingly serious environmental challenges, which can be partly addressed by their interconversion into valuable compounds. It is hypothesized that the porosity and acidity of a zeolite‐based catalyst will affect the selectivity and effectiveness, enabling a controllable and selective conversion of polyethylene (PE) into gas‐diesel or lubricating base oil. A series of embryonic, partial‐ and well‐crystalline zeolites beta with adjustable porosity and acidity are prepared from mesoporous SBA‐15. The catalysts and catalytic systems are studied with nuclear magnetic resonance (NMR), X‐ray diffraction (XRD), and adsorption kinetics and catalytic reactions. The adjustable porosity and acidity of zeolite‐beta‐based catalysts achieve a controllable selectivity toward gas‐diesel or lubricating base oil for PE cracking. With a catalyst with mesopores and appropriate acid sites, a fast escape and reduced production of cracking of intermediates are observed, leading to a significant fraction (88.7%) of lubricating base oil. With more micropores, a high acid density, and strong acid strength, PE is multiply cracked into low carbon number hydrocarbons. The strong acid center of the zeolite is confirmed to facilitate significantly the activation of hydrogen (H_2_), and, an in situ ammonia poisoning strategy can significantly inhibit hydrogen transfer and effectively regulate the product distribution.

## Graphical abstract

The reserved mesopores ensured the entry of macromolecular reactants and made full use of the acidic centers near the exposed microporous windows to realize the precise shearing of long‐chain polyethylene wastes, which enabled controllable access to diesel or lubricating oil base oils, and realized efficient and controllable conversion of macromolecular and ultra‐macromolecular wastes by zeolite‐based solid acid catalysts.

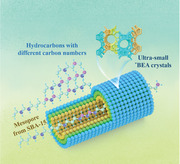

## 1 Introduction

Plastics are indispensable in modern life but their use is coupled with environmental challenges associated with waste handling.^[^ ^1^ ^]^ One recent example is the increased use of single‐use plastics during the Corona Virus Disease 2019 pandemic.^[^ ^2^ ^]^ When it comes to plastic waste, more than 60% of the overall plastic content of municipal solid waste consists of polyolefins – polypropylene (PP) and polypropylene (PE). PE has an annual production over 100 million metric tons worldwide. It is also comprised of strong C−C and C−H bonds, and as it has mainly secondary carbons, it is also highly resistant to oxidation.^[^ ^3^ ^]^ The breaking of the strong C–C single bonds in PE requires high energy, and depolymerization processes are thermodynamically challenging. Additionally, the typical depolymerization reactions are also accompanied by side‐reactions, and unwanted by‐products, ultimately resulting in a reduced recycling efficiency.^[^ ^4^ ^]^

The economic viability of waste recycling depends on if the products can offset the costs of recycling, sorting, and processing.^[^ ^5^ ^]^ Chemical methods for plastic recycling have attracted recent attention., E.g., Liu et al. prepared short‐chain wax by pyrolysis of PE, PP, and their mixtures by a thermolysis technique, and the wax was processed into a plastic‐based soap.^[^ ^6^ ^]^ Similarly, Huber et al. prepared olefin‐rich pyrolysis oils, which they upgraded by hydroformylation to aldehydes that could be hydrogenated into alcohols.^[^ ^7^ ^]^ PE and PP waste hold promise for the production of monomers, fuels, lubricating base oil, etc. Base oils comprise, the main part of lubricants. They can potentially be produced from waste PE and PP but chemical details and circularity aspects need to be further studied and developed.

Thermal‐ and catalytic or hydrocracking.^[^ ^8^ ^]^ can be used for PE depolymerization. Thermal cracking is performed at temperatures typically > 500 °C, and the energy use is high.^[^ ^8^ ^]^ The product composition is complex due to the radical mechanisms involved in the cracking.^[^ ^9^ ^]^ Catalytic cracking follows a carbenium‐ion mechanism and allows the use of far lower temperatures than thermal cracking.^[^ ^10^ ^]^ However, it typically involves the use of noble metals and sometimes hydrogen (H_2_), which increases the costs. During catalytic cracking, long‐chain hydrocarbons are adsorbed on Brønsted acid sites, protonated, and converted into carbonium‐ions that are cracked into alkane and carbenium ions.^[^ ^11^ ^]^ Carbenium ions form isomerized carbenium ions through intramolecular hydrogen transfer. Then they are subjected to four types of *β*‐scissions and produce shorter carbenium ions and *n*‐ or *iso*‐alkenes, all of the above are affected by the structure of reactants and products.^[^ ^12^ ^]^ The newly formed alkane molecules may not be desorbed rapidly, but they can still be adsorbed on Brønsted acid sites again, thus producing new carbonium ions. Based on this, polyolefin molecules may undergo multiple *β*‐scissions until low molecular weight hydrocarbons are obtained.^[^ ^13^ ^]^ The product distributions are strongly affected by the diffusion of carbenium ions, intermediates, competitive adsorption (desorption), and reaction.^[^ ^8^, ^14^ ^]^ This suggests that products with different compositions could be collected during the conversion of the polyolefins by designing and optimizing the catalyst or controlling the reaction process.

For the catalytic cracking of polyolefins, strongly acidic catalysts generate alkylbenzene and gaseous hydrocarbon through end‐cracking, aromatization, and dehydrogenation, while moderately strong acid catalysts generate liquid fractions through random‐chain cracking.^[^ ^15^ ^]^ However, strongly acidic catalysts also lead to coking. Therefore, ideal catalysts for waste plastics cracking into lubricating base oils should have suitable acidity and as will be discussed a suitable pore size distribution. Zeolites are crystalline and microporous aluminum silicates and widely used as heterogeneous catalysts and adsorbents for gas separation.^[^ ^16^ ^]^ Micropores are < 2 nm, mesopores 2–50 nm, and macropores > 50 nm in pore diameter. When the size of the guest molecules is larger or close to the size of the pore windows in the zeolite, severe diffusion restrictions occur.^[^ ^12^, ^17^ ^]^ Strategies to circumvent the diffusion limitations include increasing diffusion rates of intermediate products (liquid fractions), improving accessibility to the acid sites, and reducing secondary cracking.^[^ ^13^ ^]^ This can be implemented by using ultrasmall zeolite crystals.^[^ ^18^ ^]^ nano‐sized crystals, and so‐called hierarchical zeolites where meso‐ and/or macropores are connecting in a topologically arranged network of inter‐ or intracrystalline structures.^[^ ^19^ ^]^ Another promising route to impair diffusion limitation and improve accessibility to acid sites can be based on the use of so‐called embryonic zeolites.^[^ ^18^, ^20^ ^]^ These are related to the amorphous zeolites studied by Corma and Díaz‐Cabañas.^[^ ^21^ ^]^ These X‐ray amorphous compounds, harvested in the early stages of zeolite synthesis, have more accessible acid sites^[^ ^18c^ ^]^ than typical zeolites and displayed excellent catalytic performances in the conversion of bulky molecules such as 1,3,5‐triisopropylbenzene,^[^ ^20c^ ^]^ PP,^[^ ^18c^ ^]^ low‐density PE (LDPE).^[^ ^20a^ ^]^

**Scheme 1:**
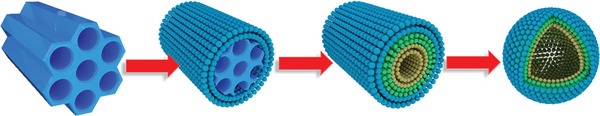
The transformation process of mesoporous SBA‐15 to embryonic beta zeolite.

Embryonic ZSM‐5 has shown prospects for the processing of polyolefins,^[^ ^18^, ^20^ ^]^ but, as far as we know, catalytic cracking of PE on embryonic beta zeolite has not been reported. The pore openings in zeolite beta are larger than in ZSM‐5 allowing a faster diffusion of the reactants, intermediates, and products, and, we hypothesize that this will positively influence the catalytic performance. Zeolite beta of the polymorph C (with the Framework Type Code BEC) has a three‐dimensional network in which a molecule of a maximum (spherical size) of 0.61 nm can diffusively translate.^[^ ^22^ ^]^ Polyolefin cracking over a large‐pore zeolite such as the zeolite beta will generate a larger fraction of liquid products than small‐ and medium‐pore size zeolites.^[^ ^23^ ^]^ In this study, we prepared embryonic beta zeolites with adjustable porosity and acidity from mesoporous silica SBA‐15.^[^ ^24^ ^]^ via a solid conversion route (**Scheme** **1**), and tested if residual mesoporosity would facilitate improved mass transfer and acid accessibility during the catalytic cracking of PE into gasoil, diesel, or lubricating base oil.

## 2 Results and Discussion

### 2.1 Characteristics of the Zeolitic Beta Catalysts

**Figure 1:**
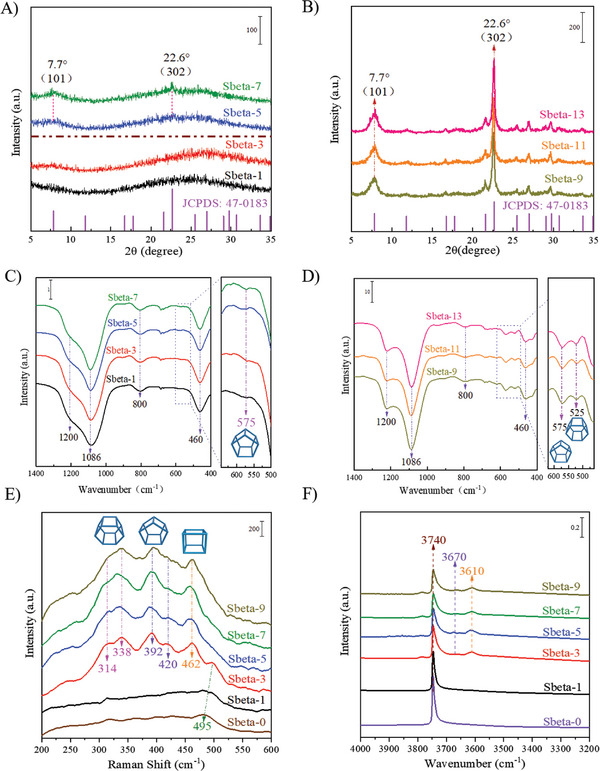
A,B) XRD patterns. C,D) IR spectra. E) UV‐Raman spectra. F) In situ DR‐IR spectra of embryonic and well‐crystallized zeolite Sbeta‐t, “t” is the crystalline time, h.

Zeolite beta‐containing catalysts were synthesized with an SBA‐15 silica source and from analysis of the X‐ray diffraction (XRD) patterns it was clear that more zeolite beta developed for longer time of crystallization (**Figure** **1**A,B). Samples Sbeta‐t (t ≤ 3) did not diffract X‐rays and were X‐ray amorphous.^[^ ^18^, ^21^ ^]^ They scattered X‐rays, with peaks attributed to the closest and second‐closest neighboring atoms of the structures.^[^ ^18^, ^20^ ^]^ It can be noted that XRD analysis is not suitable to analyze the structure of ultra‐small zeolite crystals with short‐range order.^[^ ^25^ ^]^ Sbeta‐t (t ≥ 9) samples have characteristic reflections of the beta‐type topology (JCPDS No. 47–0183) at 2θ = 7.6 ° and 22.6 ° corresponding to (101) and (302) crystal planes. No diffraction peaks belonging to other impurity crystalline phases were observed. Samples Sbeta‐t (t ≥ 9) were pure zeolite beta. The infrared spectra (IR) of the as‐made samples in Figure 1C,D have three intense absorption bands around 1086, 800, and 460 cm^−1^ corresponding to asymmetric Si‐O, symmetric Si‐O stretching vibrations and the Si‐O‐Si bending mode.^[^ ^18^, ^26^ ^]^ The vibrational bands around 1200 cm^−1^ are affected by linkages between (Si, Al)O_4_ tetrahedra, and these become more defined for the crystalline Sbeta‐t (t ≥ 9), consistent with increased crystallinity. The vibrational bands around 575 cm^−1^ and 525 cm^−1^ belong to double five‐membered rings (D5R) and double six‐membered rings (D6R), respectively. The band for D6R developed for the highly crystalline samples Sbeta‐t (t ≥ 9). The vibration peaks around 300–350 cm^−1^, 370–440 cm^−1^ and 450–470 cm^−1^ in the ultraviolet (UV) Raman spectra (Figure 1E) correspond to D6R, D5R, and double four‐membered rings (D4R),^[^ ^27^ ^]^ of zeolite beta, and were detected for Sbeta‐t (t ≥ 3), while the intensities for the asymmetric stretching vibration of the Si─O─Si bond (485–515 cm^−1^).^[^ ^28^ ^]^ are successively reduced in intensity, consistent with the growth of zeolite nanocrystals. From analysis of the in situ diffuse reflectance‐IR (DR‐IR) spectra in Figure 1F, it is clear that the SBA‐15 and Sbeta‐1 have an isolated silicon hydroxyl peak at 3740 cm^−1^, while the hydroxyl infrared peaks on Sbeta‐t (t ≥ 3) are broad and complex. The peaks at around 3500–3700 cm^−1^ are tentatively assigned to the Al hydroxyl group, for example, the vibration bands at 3610 cm^−1^ and 3670 cm^−1^ are ascribed to the bridged hydroxyl group and extra‐framework aluminum hydroxyl.^[^ ^29^ ^]^ With this information it was clear that some zeolite beta framework had been created in Sbeta‐t (t ≥ 3), suggestively possesses a regular zeolite framework. This analysis is consistent with that Sbeta‐3, has some Brønsted acid sites, and while still being X‐ray amorphous. The exact transition into well‐developed crystals is difficult to detected due to the rapid growth of crystals after the induction period.^[^ ^26b^ ^]^

The nitrogen (N_2_) adsorption–desorption isotherm (Figure S1A, Supporting Information) for the Sbeta‐1 is classified as an apparent type IV isotherm (mesoporous material with an H1‐type hysteresis loop caused by capillary condensation), and the pore size distribution (PSD) (Figure S1B, Supporting Information) for Sbeta‐1 has a uniform pore size centered at 6.9 nm. The pristine SBA‐15 silica has a maximum at about 11 nm (Figure S2B, Supporting Information). For Sbeta‐t (t ≥ 3), the mesopores are centered at around 2.5 nm (Figure S1B, Supporting Information). The isotherms of Sbeta‐t (1 < t ≤ 7) are combinations of type I (micropores) + IV (mesopores). The mesoporous volumes (**Table** **1**) are reduced with the crystallization time, and the microporous volumes increased. For Sbeta‐t (t ≥ 9), the uptake at high pressures (P/P_0_ = 0.9) is high. This analysis shows that mesopores shrunk significantly with the formation of Sbeta‐X, which was tentatively assigned to nanosized crystals accumulation in the pores.^[^ ^30^ ^]^ In a study investigating the pore structure characterization of samples with varying crystallization durations, it is observed that the crystallization time exhibited a strong correlation with crystal development. Notably, the manipulation of crystallization time enabled precise regulation of the pore structure in the catalysts, thereby highlighting the enhanced controllability of the solid‐phase crystallization approach. Combined with the results presented in Table 1 and Figure S1B (Supporting Information), it is confirmed that a series of well‐crystallized Sbeta‐t (t ≥ 9) zeolites with pore size distribution around 2 to 80 nm can be obtained, reflecting that the pore structure of the as‐synthesized samples can be tailored by controlling the synthesis time.

**Table 1:** Pore structure parameters of as‐prepared catalysts.

Samples^a^ ^)^	S_BET_ [m^2^ g^−1^]	S_micro_ [m^2^ g^−1^]	S_ext_ [m^2^ g^−1^]	V_micro_ [m^3^ g^−1^]	V_meso_ [m^3^ g^−1^]
Sbeta‐0	565	15	550	0.01	1.15
Sbeta‐1	434	18	416	0.01	0.71
Sbeta‐3	315	70	245	0.03	0.50
Sbeta‐5	202	82	120	0.03	0.35
Sbeta‐7	193	86	107	0.05	0.28
Sbeta‐9	699	584	115	0.23	022
Sbeta‐11	694	588	106	0.23	0.23
Sbeta‐13	790	705	85	0.28	0.16

^a)^ The number in the sample name stands for the time used for the synthesis of the zeolite beta catalysts.

**Figure 2:**
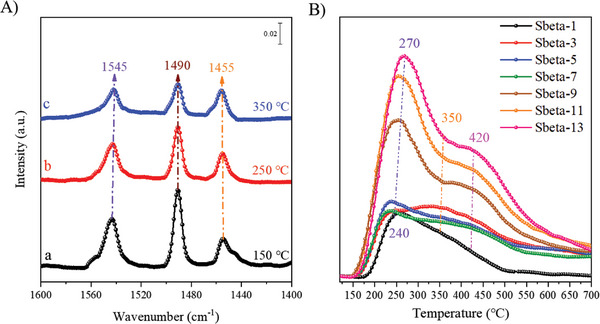
A) IR spectra of pyridine adsorbed on Sbeta‐3 sample. B) NH_3_‐TPD curves of embryonic and well‐crystallized zeolite beta. The number denotes the time of synthesis.

The zeolite‐beta containing catalysts were prepared in their H‐forms. Due to the defect structures in zeolite beta, this zeolite has internal Lewis acid groups and external Brønsted acid sites groups,^[^ ^21^ ^]^ and these can be quantified by IR adsorption of pyridine. As shown in **Figure** **2**A, three bands are detected for adsorbed pyridine on the Sbeta‐3 sample, at the three studied temperatures. The peak at 1455 cm^−1^ is attributed to pyridine adsorbed on Lewis acid sites, and the one at 1545 cm^−1^ to protonated pyridine on Brønsted acid sites.^[^ ^31^ ^]^ The peak at 1490 cm^−1^ is usually assigned to pyridine adsorbing simultaneously on Brønsted and Lewis acid sites.^[^ ^32^ ^]^ The intensity of the bands for Lewis acid sites barely changed on an increasing desorption temperature but the intensity for the Brønsted acid sites decreased, consistent with a higher heat of adsorption on the Lewis acid sites is higher. Even with the desorbed temperature increasing to 350 °C, Lewis acid sites slightly decrease from 26 to 22 µmol g^−1^; while Brønsted acid sites decrease significantly from 62 to 33 µmol g^−1^ (Table S1, Supporting Information). As shown in Figure 2B, all samples exhibit ammonia (NH_3_) desorption peaks: one low temperature peak at ≈250 °C is assigned to the desorbed NH_3_ from weak acid sites, one at ≈350 °C is assigned to the desorbed NH_3_ from medium acid sites, and the last one at about ≈420 °C is ascribed to the NH_3_ desorption from strong acid sites.^[^ ^32^ ^]^ The strong acid sites are typically assigned to Lewis acid sites.^[^ ^33^ ^]^ From the features in Figure 2B it is clear that peaks corresponding to weak or strong acid sites are high in intensity for Sbeta‐t (t ≥ 9). This enhanced intensity is consistent with the regularity of the corresponding zeolite crystals as supported by also analyses of XRD patterns (Figure 1A,B), UV‐Raman spectra (Figure 1E) and in situ DR‐IR spectra (Figure 1F).

**Figure 3:**
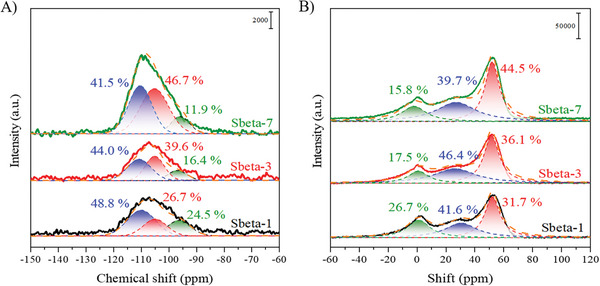
A) ^29^Si MAS NMR spectra of Sbeta‐1, Sbeta‐3 and Sbeta‐7 samples. B) ^27^Al MAS NMR spectra of Sbeta‐1, Sbeta‐3 and Sbeta‐7 samples. The number in the sample name stands for the time used for the synthesis of the zeolite beta catalysts.

The relative proportions of Si and Al in different coordination environments for Sbeta‐t (t ≤ 7) samples were estimated by regression analysis of the corresponding solid state ^29^Si and ^27^Al magic‐angle spinning nuclear magnetic resonance (MAS NMR) spectra.^[^ ^34^ ^]^ with data in **Figure** **3**A,B, Tables S2 and Figure S3 (Supporting Information). The signals around −110 ppm in the ^29^Si MAS NMR spectra were attributed to Q^4^ [Si(4Si, 0Al)] species; those around −105 ppm to the Q^3^ [Si(3Si, 1Al)] species and those around −95 ppm to Q^2^ [Si(2Si, 2Al)] species.^[^ ^35^ ^]^ The fraction of Q^4^ species decreased from 48.8% (Sbeta‐1) to 44.0% (Sbeta‐3), and to 41.5% (Sbeta‐7), and Q^2^ species decreased from 24.5% (Sbeta‐1) to 16.4% (Sbeta‐3), and to 11.9% (Sbeta‐7). The fraction of Q^3^ species correspondingly increased from 26.7% (Sbeta‐1) to 39.6% (Sbeta‐3), and to 46.7% (Sbeta‐7). These data were consistent with that SiO_2_ depolymerized and condensed with Al hydroxyls. The gradual conversion into Q^3^ species with increasing crystallization tune is consistent with the entry of Al species into the zeolite beta framework (Table S2, Supporting Information). The ^27^Al shift at around 0 ppm was assigned to hexa‐coordinated aluminum (Al^VI^, called as extra‐framework aluminum (EFAl).^[^ ^36^ ^]^ The ^27^Al peak at around 30 ppm was tentatively attributed to penta‐coordinated aluminum. The band at around 52 ppm was classified as tetra‐coordinate aluminum (Al^IV^, called as framework aluminum (FAl)).^[^ ^37^ ^]^ The relative fraction of Al^VI^, as determined from the integral intensities (Table S3 and Figure S3, Supporting Information) decreased from 26.7% (Sbeta‐1) to 17.5% (Sbeta‐3), and to 15.8% (Sbeta‐7), while the fraction of Al^V^ increased from 41.6% (Sbeta‐1) to 46.4% (Sbeta‐3), and then decreased to 39.7% (Sbeta‐7). The fraction of Al^IV^ increased from 31.7% (Sbeta‐1) to 36.1% (Sbeta‐3), and to 44.5% (Sbeta‐7). The result is consistent with that EFAl transformed primarily to Al^V^ species not in the framework, which polycondensated with adjacent silicon hydroxyl into FAls. It is noted that Al^V^ has gotten great attention as a potential candidate for increasing the acidity.^[^ ^39b,c^ ^]^ Al^IV^ is the main source of Brønsted acid sites after being exchanged by ammonium ions followed by calcination.

**Figure 4:**
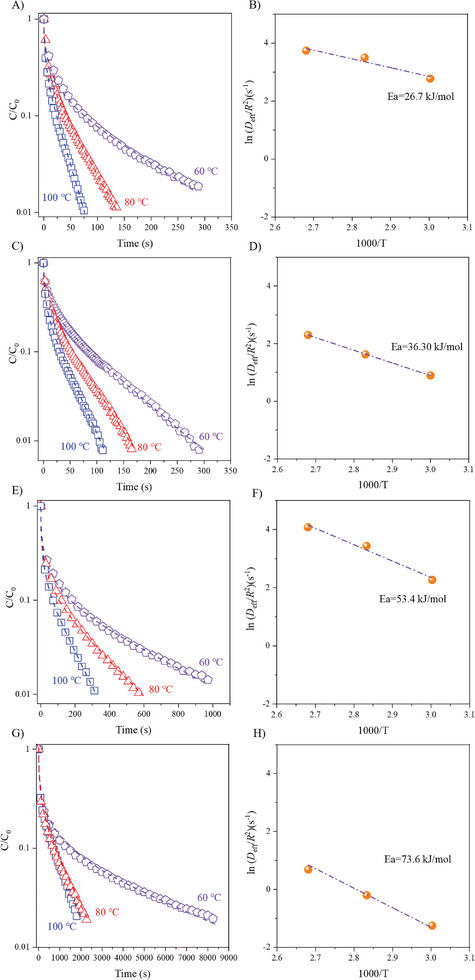
A) The diffusion curves of toluene on Sbeta‐3. B) The relationship between *D*_eff_/*R* ^2^ and temperatures on Sbeta‐3. C) The diffusion curves of toluene on Sbeta‐5. D) The relationship between *D*_eff_/*R* ^2^ and temperatures on Sbeta‐5. E) The diffusion curves of toluene on Sbeta‐7. F) The relationship between *D*_eff_/*R* ^2^ and temperatures on Sbeta‐7. G) The diffusion curves of toluene on Sbeta‐9. H) The relationship between *D*_eff_/*R* ^2^ and temperatures on Sbeta‐9. The number in the sample name stands for the time used for the synthesis of the zeolite beta catalysts.

Zero Length Column (ZLC) and its time dependent desorption curves are presented for toluene in **Figure** **4**, and the experimental data coincides well with the regression analysis. This match indicates that the so called Crank diffusion model is applicable to the experimental results.^[^ ^38^ ^]^ of strongly adsorbed toluene.^[^ ^39^ ^]^ The apparent diffusion rate (*D*_eff_/*R* ^2^) at different temperatures and corresponding diffusion activation energies are calculated and are listed in **Table** **2**, with *R* being the radius of the particles. For four samples, the *D*_eff_/*R* ^2^ of toluene accelerates with the increasing desorption temperatures, due to the strong temperature dependence of the diffusion.^[^ ^40^ ^]^ The kinetic diameter of toluene (0.58 nm).^[^ ^41^ ^]^ is smaller than the microporous diameter of zeolite beta (0.67 nm), which indicates that a reduced *R* may give a larger *D*_eff_/*R* ^2^. The acidity in the micropores is also expected to reduce the diffusion, because of a cation π interaction between Brønsted acid centers and the toluene.^[^ ^42^ ^]^ The *D*_eff_/*R* ^2^ decrease significantly for samples prepared with a long crystallization time (Table 2), and the diffusion activation energy is enhanced for the more crystallized samples. These trends are consistent with the crystals gradually becoming larger, the nature of the acid properties changing,^[^ ^43^ ^]^ and the remaining mesoporosity is reduced. (The experimental regression parameter (*L*) is more > 5, indicating that the diffusion process is kinetically controlled.^[^ ^44^ ^]^)

**Table 2:** The diffusion parameters of embryonic and well‐crystallized zeolite beta.

Samples^a^ ^)^	*T* [°C]	*L*	*β*	*D*_eff_/*R* ^2^ [×10^−4^ s^−1^]	*Ea* [kJ mol^−1^]
	60	7.29	2.73	16.0	
Sbeta‐3	80	5.49	2.61	33.0	26.69
	100	7.54	2.74	42.0	
	60	16.31	2.95	9.65	
Sbeta‐5	80	7.79	2.76	31.0	36.30
	100	7.26	2.73	59.0	
	60	18.6	2.97	2.45	
Sbeta‐7	80	16.0	2.94	5.10	53.38
	100	13.1	2.91	10.0	
	60	21.0	2.99	0.212	
Sbeta‐9	80	9.83	2.83	1.35	73.58
	100	7.75	2.75	1.80	

^a)^ The number in the sample name stands for the time used for the synthesis of the zeolite beta catalysts.

**Figure 5:**
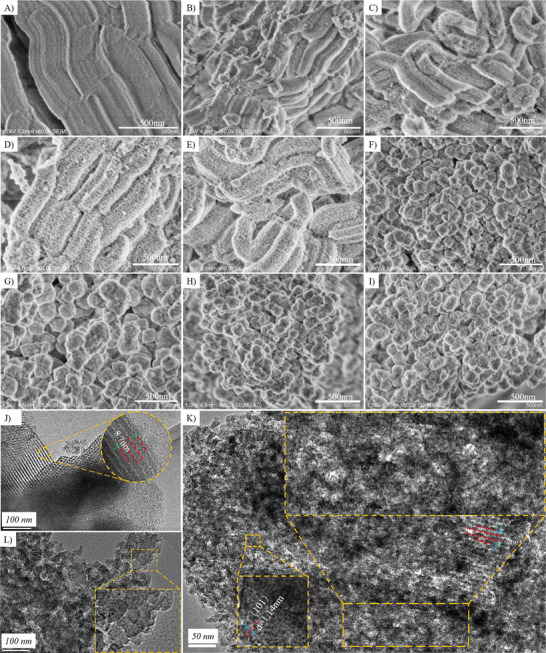
SEM images of samples. A) SBA‐15. B) Sbeta‐1. C) Sbeta‐3. D) Sbeta‐5. E) Sbeta‐7. F) Sbeta‐9. G) Sbeta‐11. H) Sbeta‐13. I) Sbeta‐15. TEM images of samples. J) Sbeta‐1. K) Sbeta‐3. L) Sbeta‐5. The number in the sample name stands for the time used for the synthesis of the zeolite beta catalysts.

Analysis of the scanning electron microscopy (SEM) and transmission electron microscopy (TEM) images in **Figure** **5** shows that the morphologies and crystal sizes of the as‐synthesized samples are intensely affected by the crystallization process. Sbeta‐t (t ≤ 7) maintained the crooked tube morphology of pristine SBA‐15 (Figure 5A) in a similar manner as was observed by Gao et al.^[^ ^45^ ^]^ Sbeta‐t (t ≤ 7) with extended crystallization time, the surfaces of the samples became rougher, and many “small opening” were observed on the surfaces, and nano‐particles were visible on Sbeta‐3, −5, and −7. The nano‐sized particles in Sbeta‐9 were the largest. They comprised loosely polycrystalline aggregates of primary nanocrystals with an average diameter of about 20 nm, consistent with a Scherrer analysis (Table S4, Supporting Information).^[^ ^46^ ^]^ TEM analysis (Figure S4A, Supporting Information) showed that the diameter of the channels in the SBA‐15 was close to 15 nm, and consistent with the 11.2 nm from N_2_ density functional theory (DFT) analysis (Figure S2B, Supporting Information). The characteristic mesoporous diameter in Sbeta‐1 and Sbeta‐3 were 8.7 nm and 7.7 nm, and lattice stripes from embryo microcrystals were observed in high‐resolution TEM images of sample Sbeta‐3 (Figure 5K). The *d*‐spacing (1.1 nm) derived from lattice stripes corresponded to the (101) crystal plane of zeolite beta crystal.^[^ ^47^ ^]^ For Sbeta‐5, −7, and −9, similar information was observed by SEM, and in Sbeta‐9, nano‐sized particles with a diameter of about ≈20 nm could be clearly observed (Figure S4C, Supporting Information). Consistently, it seems as the accumulated nano‐particles are ultra‐fined zeolite beta crystals and that the rapid crystal growth occurred in the period of 7–9 h after the start of synthesis. A gradual transformation of SBA‐15 to partially crystallized zeolite beta was consistent with SEM, TEM, IR, UV‐Raman, and N_2_ adsorption‐desorption analyses. In general, by controlling the crystallization time so as to enable precise control over crystal development, a series of embryonic and well‐crystalline zeolite beta with different porosity and crystallinity were prepared, and these X‐ray amorphous materials have Brønsted acid and Lewis acid sites.

### 2.2 Catalytic Performance

The Sbeta‐t (3 ≤ t ≤ 9) were studied as catalysts for cracking of PE into molecules with different chain lengths in N_2_ and H_2_ atmospheres. In a control experiment, carbon tatrachloride (CCl_4_) was heated to the reaction temperature together with the catalyst (Sbeta‐9) in an H_2_ atmosphere (4 MPa) and a large amount of hydrogen chloride (HCl) appeared in the gaseous products. (They also contained minor quantities of methane (CH_4_) and significant quantities of trichloromethane (CHCl_3_), dichloromethane (CH_2_Cl_2_) and monochloromethane (CH_3_Cl) after reaction), and the liquid product was detected as strongly acidic by pH indicator strips, which confirmed the strong hydrogenation activity; the acid centers in Sbeta‐9 were able to directly activate H_2_ and the hydrodechlorination of CCl_4_. To avoid the influence of heteroatoms such as halogens on the reaction, cyclohexane was chosen as the solvent, and stability experiments confirmed that cyclohexane did not react under the conditions used. Zeolite beta was chosen because it has large 12‐ring openings and a 3‐dimensional structure.^[^ ^35^ ^]^

The performance of PE thermal cracking in the absence of a catalyst, including conversion, carbon balance, and liquid product yield, is depicted in Figure S5 (Supporting Information). Notably, the PE conversion was mere 1.8%, while the yield of gaseous products was negligible, warranting no further analysis. The liquid products were predominantly concentrated in the C_50_^+^ range, with an overall liquid yield of just 1.4%. This underscores the significant challenge in achieving effective thermal cracking of PE without the aid of a catalyst. Therefore, we can confidently dismiss the potential impact of thermal cracking on subsequent catalytic cracking and hydrocracking reactions. In addition, experiments on reaction temperature, catalyst/PE ratio and reaction time were also carried out for determining the optimized reaction conditions, and the results are given in Figure S6 (Supporting Information). It can be found that in order to achieve the complete conversion of PE over the four catalysts, the optimized reaction conditions were a reaction temperature of 260 °C, a catalyst /PE ratio of 1 and a reaction time of 240 min.

#### 2.2.1 Catalytic Cracking of PE

**Figure 6:**
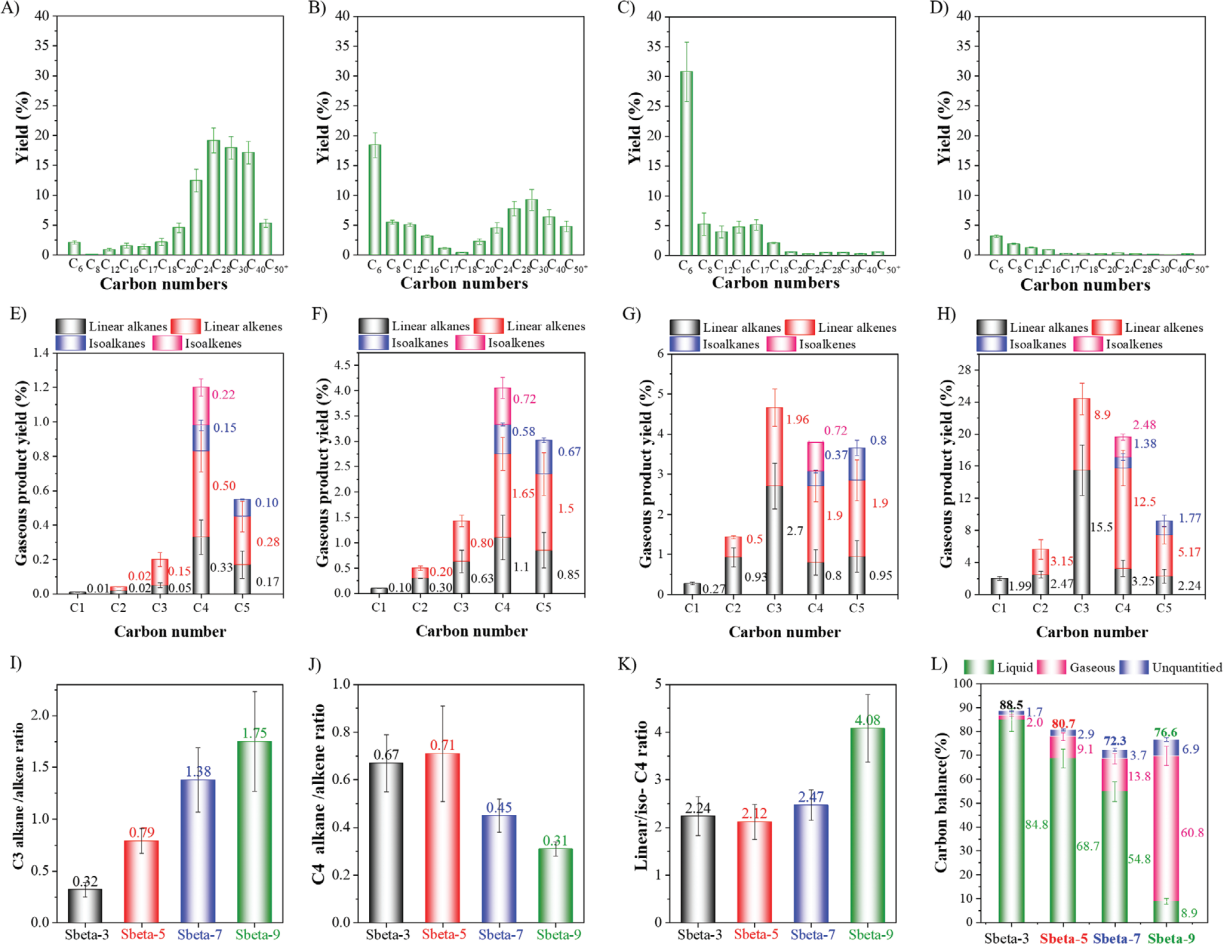
The liquid products distribution of catalytic cracking of PE reaction over different catalysts. A) Sbeta‐3. B) Sbeta‐5. C) Sbeta‐7. D) Sbeta‐9. Corresponding gaseous products distribution. E) Sbeta‐3. F) Sbeta‐5. G) Sbeta‐7. H) Sbeta‐9. I) The ratio of C_3_ alkanes/alkenes. J) The ratio of C_4_ alkanes/alkenes. K) The ratio of linear/*iso*‐C_4_. L) The product's carbon balance over four catalysts. The number in the sample name stands for the time used for the synthesis of the zeolite beta catalysts. Reaction conditions: 200 mg of PE, 20 g of cyclohexane and 200 mg of catalyst; 2.0 MPa N_2_ atmosphere, 500 rpm, 260 °C, 240 min.

**Figure 7:**
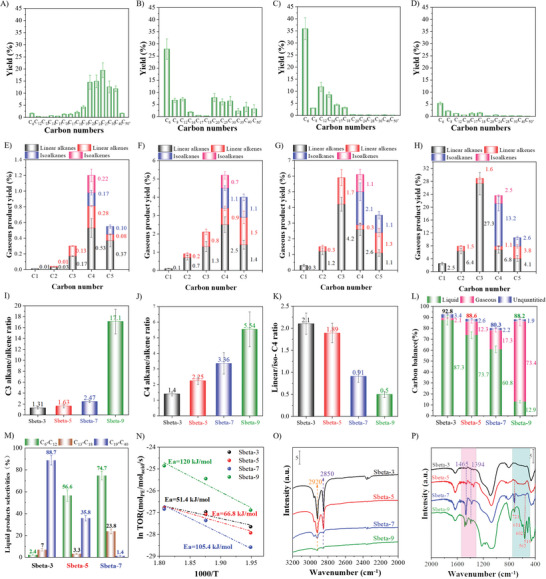
The liquid products distribution of hydrocracking of PE reaction over different catalysts. A) Sbeta‐3. B) Sbeta‐5. C) Sbeta‐7. D) Sbeta‐9. Corresponding gaseous products distribution. E) Sbeta‐3. F) Sbeta‐5. G) Sbeta‐7. H) Sbeta‐9. I) The ratio of C_3_ alkanes/alkenes. J) The ratio of C_4_ alkanes/alkenes. K) The ratio of linear/*iso*‐C_4_. L) The product's carbon balance over four catalysts. M) Selectivity of gasoline, diesel, and lubricating base oils on Sbeta‐3, Sbeta‐5, and Sbeta‐7 catalysts. N) The relationship between turnover rates and reaction temperatures. O) IR spectra of residual PE on the four catalysts after reaction. P) IR spectra of coke on four catalysts after reaction. The number in the sample name stands for the time used for the synthesis of the zeolite beta catalysts. Reaction conditions: 200 mg of PE, 20 g of cyclohexane and 200 mg of catalyst; 2.0 MPa H_2_ atmosphere, 500 rpm, 260 °C, 240 min.

Information on the yields, and alkane‐to‐alkene ratios on the cracking of PE with Sbeta‐t (3 ≤ t ≤ 9) in N_2_ are shown in **Figure** **6**. At 260 °C and 2 MPa of N_2_, the PE was completely catalytically cracked within 4 h, while the selectivity of the final products was dependent on the version of zeolite beta used. For the yields of the final cracking liquid products (see Figure 6A–D), the main products were C_24_ to C_40_ for Sbeta‐3, and with Sbeta‐9 mainly gaseous products were observed. The gaseous product distributions (see Figure 6E–H) from cracked PE on Sbeta‐3 and Sbeta‐5 consisted of mainly of C_4_ and C_5_ hydrocarbons, while on Sbeta‐7 and Sbeta‐9 it included also C_1_, C_2_, and C_3_ hydrocarbons. With crystalline zeolite beta the C_2_ and C_3_ fractions increased. Significant amounts of ethylene and propylene were observed over the Sbeta‐9 catalyst. The hydrogen transfer index (HTI) of the C_3_ and C_4_ products are illustrated in Figure 6I,J, and the HTI was high (1.75) for the C_3_ products on Sbeta‐9. For C_4_ products it was significantly smaller than that of C_3_ products, which was caused by that C_4_ hydrocarbons could still be cracked to C_3_ and methane on strong acid centers.^[^ ^48^ ^]^ The high HTI results for C_3_ products of Sbeta‐9 indicated that a more graphitized coke would be produced, as the reduced mesoporous volume and stronger acidity would make it more susceptible to deactivation during the reaction process (cf. Table 1 and Figure 2B). The ratios of linear C_4_ and *iso*‐C_4_ (shown in Figure 6K) were similar for Sbeta‐3, Sbeta‐5 and Sbeta‐7, and increased significantly for Sbeta‐9. It seems as the crystalline Sbeta‐9 did not have sufficient amounts of residual weak acid sites to effectively support isomerization of the linear‐C_4_ product. The carbon balances of the cracking of PE (shown in Figure 6L) were the highest for Sbeta‐3 and the lowest for Sbeta‐7. The reduction for Sbeta‐7 was related to that it converted PE partly to C_5_ and C_6_ hydrocarbons that in turn led to evaporation and problems in collecting the liquid products, leading to a large error in the analysis process (Supporting Information has confirmed the result). Sbeta‐9 catalyst with its high acidity and crystallinity, gave a product distribution that was more accurately analyzed, as the C_5_ and C_6_ were converted to gaseous products C_3_ and C_4_. In addition to liquid and gaseous products, a minor accumulation of coke on the spent catalysts led to a diminished carbon balance. This coke primarily comprised paraffinic and aromatic hydrocarbon species. It can be inferred from **Figure** **7**P that with the increased crystallinity in the catalysts, aromatic coke deposition increased; on the contrary, with the decreased crystallinity, coke deposition with aromatic property decreased while the one with aliphatic group increased. It can be noted that the zeolite beta with strong acidic sites and high crystallinity (such as Sbeta‐9) readily cracked the PE into small molecules.^[^ ^49^ ^]^ With increasing synthesis time, the zeolite beta catalyst displayed a reduced mesoporosity, increased acid density and a stronger acidity.^[^ ^15^, ^50^ ^]^

DFT calculations indicated that lower carbon numbers of the reactants corresponded with a higher energy barrier of cracking reactions, and that the reaction energy barrier of the end‐cracking was higher than that of middle‐cracking, which means that diffusion‐limited catalysts with high crystallinity were more inclined to undergo end‐cracking at strong acid sites, and a large number of small molecules of gaseous hydrocarbons are obtained eventually (Figure S7 and Table S5, Supporting Information). This is consistent with that stronger acid centers (corresponding to lower deprotonation energies) in the Sbeta catalysts lead to a lower energy barrier for the hydrocarbon cracking reaction.^[^ ^51^ ^]^ In addition, the textural nature and acid density of the Sbeta catalysts exhibited a notable correlation with their catalytic performance, as demonstrated in Supplementary Information (Figure S8, Supporting Information). Approximately linear relationships were observed between the properties of the Sbeta catalysts and various metrics such as the gaseous and liquid product yields, as well as the HTI. Specifically, an increase in the mesoporous volume of the Sbeta catalyst led to an enhancement in the yield of liquid products, whereas an increase in the microporous volume resulted in a higher yield of gaseous products. Furthermore, a high acid density favors the production of gaseous products, leading to a decrease in the yield of liquid products. Notably, an augmentation in the microporous volume enhanced hydrogen transfer for C_3_ products, yet induced cleavage of C_4_ products, deviating from hydrogen transfer and isomerization reactions within the micropores. Finally, a positive correlation was established between the effective acid centers on the outer surface of the Sbeta catalyst and the cracking of PE to liquid products.

#### 2.2.2 Hydrocracking of Polyethylene

The hydrocracking cracking of PE with Sbeta‐t (3 ≤ t ≤ 9) was tested under the same conditions as the cracking but with H_2_ instead of N_2_ atmosphere. For the liquid products, the carbon number distribution (Figure 7A–D) was largely similar to those under N_2_ atmosphere. For the gaseous products (Figure 7E–H), the most obvious difference was that the relative content of olefins was reduced significantly under H_2_ atmosphere. The olefins underwent hydrogenation into alkanes.^[^ ^52^ ^]^ E.g., the gaseous product distributions of Sbeta‐7 samples under N_2_ atmosphere $C_{2}^{=}$ 0.5%, C_2_ 0.93%, $C_{3}^{=}$ 1.96%, C_3_ 2.7% while under H_2_ atmosphere $C_{2}^{=}$ 0.3%, C_2_ 1.2%, $C_{3}^{=}$ 1.7%, C_3_ 4.2%). The “HTI' of the C_3_ products (Figure 7I,J) increased significantly under H_2_ as compared to N_2_, which was attributed to that the acid centers activated H_2_ and hydrogenated the cracked olefinic product. As expected the most acidic and crystalline zeolite beta (Sbeta‐9) displayed the highest change in ‘HTIs' confirming that well developed zeolites have higher hydrotreating activity than less crystalline catalysts. Also, the ‘HTI” for the C_4_ products was higher under H_2_ than under N_2_ as was expected. It is noted that the “HTI” of Sbeta‐3 was significantly smaller than for Sbeta‐9 under H_2_ atmosphere (1.4% vs 5.54%) but higher under N_2_ atmosphere (0.67% vs 0.31%). The higher value under N_2_ for the low‐acidity catalyst (Sbeta‐3) was attributed to that the olefins produced on the high‐acidity catalyst (Sbeta‐9) in N_2_ more easily underwent Diels‐Alder addition reactions on the strong acid sites.^[^ ^53^ ^]^ Note that we used the term “HTIs' in case of H_2_ to indicate that the C_3_ and C_4_ products may include hydrogen transfer from olefins to the corresponding alkanes and hydrogenation reactions occurring by activated hydrogen. It differentiate from the term HTIs used for reactions in N_2_. The ratios of linear/*iso*‐C_4_ in the gaseous products were consistently lower in H_2_ than in N_2_ atmosphere, as seen from Figure 7K. This was related to that the acid sites enabled the activation of H_2_, which on the one hand inhibited the generation of coke prolonging the catalyst life, but also promoted dehydrogenation of hydrocarbons into olefins susceptible to isomerization reactions. The carbon balances (compare Figures 6, 7) were higher under H_2_ than under N_2_, which may be due to lower coke of the catalysts under H_2_ atmosphere because of the additional hydrogen sources.^[^ ^52^ ^]^ The relative proportions of gaseous and liquid products were similar for the two different conditions, irrespectively of which zeolite beta catalyst used. The selectivity of lubricating base oil was the highest for Sbeta‐3, and dropped when more acidic catalysts were used (Figure 7M). C_6_‐C_12_ hydrocarbons are ascribed to gasoline fraction, C_13_‐C_18_ hydrocarbons are assigned to diesel oil fraction, and C_19_‐C_40_ hydrocarbons are classified to lubricating base oil. It is also noted that Sbeta‐3 also has abundant mesopores contributing to a fast escape of the produced liquid fraction, avoiding deep cracking of intermediate products,^[^ ^10b^ ^]^ increasing the yield of the liquid fraction, and depressing the formation of light gaseous products. With the catalysts, Sbeta‐5, −7, and −9, with increasing acid strength and decreasing mesoporosity, the product distributions were tuned more and more to the short chain molecules on hydrocracking of PE. For the most acidity catalyst with little mesoporosity (Sbeta‐9) most products were gaseous (Figure 7H,L). In addition, the textural properties and acid density of the catalyst exhibit a discernible correlation with its catalytic performance, as depicted in Supplementary Information (Figure S9, Supporting Information). These correlations mirrored the trends observed during polyethylene cracking, albeit with distinct variations in the C_4_ product profiles during hydrocracking. Specifically, an augmentation in the microporous volume of the Sbeta conspicuously elevates the ‘HTIs” of the C_4_ products. This enhancement is likely attributed to the facilitation of C_4_ olefin hydrogenation within the micropores of the Sbeta catalyst, resulting in the formation of C_4_ alkanes, due to the introduction of an additional hydrogen source. Concurrently, this process also promoted the hydroisomerization of linear‐C_4_ products within the micropores, leading to the generation of a substantial quantity of *iso*‐C_4_ products.

From the temperature‐dependent turnover rates (TOR) of the hydrocracking of PE over the four Sbeta catalysts (Figure 7N), the apparent activation energies were calculated. It was the highest for Sbeta‐9 and the lowest for the Sbeta‐3. This trend was surprising as Sbeta‐9 had the highest acidity (density and strength). Sbeta‐3 had a significant mesoporosity and Sbeta‐9 not. The high activation energy on Sbeta‐9 was ascribed to kinetic control with diffusion restrictions for long‐chain PE and difficulties of accessing the micropores with their acid centers. A large mesoporosity seems to facilitate the access for the reactants, which is corroborated with the diffusion properties (cf. Figure 4). Also, the IR spectra of the Sbeta‐3 and Sbeta‐5 after the reaction (Figure 7O) display bands of a high intensity that corresponds to the –CH_2_ groups in residual hydrocarbons, which supports that a large mesoporous volume facilitates the larger hydrocarbons to diffuse and adsorb on the catalysts. From the IR spectra in another region of the spent catalysts (Figure 7P), it can be observed distinct vibrational peaks at 500–700 cm^−1^ on the spent Sbeta‐9 catalyst. According to open literature,^[^ ^54^ ^]^ the signals in this region can be caused by polysubstituted benzene species and coke formation during the catalytic cracking of PE on strongly acidic Sbeta‐9.^[^ ^15^ ^]^ Due to its large number of strong acids sites (cf. Figure 2B), linear PE is cracked into short‐chain molecules on Sbeta‐9, among which some undergo aromatization, alkyltransfer and other reactions, leading to a significant fraction of polysubstituted aromatics.^[^ ^15^ ^]^ The spent Sbeta‐9 catalyst also shows weak vibration peaks at 1300–1500 cm^−1^ in the IR spectra, which are attributed to the bending vibration of ‐CH_3_ group in molecules of a paraffinic nature.^[^ ^55^ ^]^ These results may indicate that coke with a small amount of paraffinic nature accompanied by a large amount of polysubstituted benzene exists in the used and strongly acidic Sbeta‐9.^[^ ^15^, ^50^ ^]^ The spent Sbeta‐7 catalyst had less amount of included aromatic coke, and spent Sbeta‐5 and Sbeta‐3 catalysts had almost no polysubstituted aromatic coke. Thermogravimetric‐Differential Scanning Calorimetry (TG‐DSC) curves of the used catalysts were recorded to further assess their nature and are displayed in Figure S10 (Supporting Information). At temperatures < 200 °C, the weight loss was the highest for the most crystalline catalyst Sbeta‐9 and correlated with the microporous volume. At the 200–500 °C stage, ascribed to the oxidative decomposition of coke, the DSC curves for Sbeta‐5, −7, and −9, displayed strong exothermic peaks, and the Derivative Thermogravimetry (DTG) curves a rapid rate of weight loss, indicating that the coke was removed by oxidative decomposition. The corresponding weight loss on Sbeta‐5 is 15.9% and significantly higher than the 9.7% of Sbeta‐7 and 10.7% of Sbeta‐9, respectively. Sbeta‐3 had only little mass of coke, and no significant signal peaks were detected in the DTG and DSC curves. The coke was oxidized at a lower temperature in Sbeta‐5 than in Sbeta‐7, and Sbeta‐9 (425 °C), which was consistent with its lower degree of aromatization, and that this catalyst had a higher mesoporous volume. The weight loss > 500 °C can have been caused by dehydration and condensation of silica hydroxyl groups.^[^ ^19c^ ^]^ but was not analyzed further.

Overall the data is consistent with that a zeolite catalyst with a high acid strength and density leads to a short chain length of the product molecule. With an increased acid amount and strength, there is a low deprotonation energy, and further cleaving of short‐chained molecules is expected. The Brønsted acid centers donate protons which that reduces the transition‐state levels of the involved reactions. PE is expected to be converted into carbonium intermediate species on the Brønsted acid sites and cracked into an alkane and a carbenium intermediate.^[^ ^11^ ^]^ that desorb from acid sites and form the corresponding alkene. High acid density contributes to the repeated formation of carbonium intermediate species followed by multiple *β*‐scissions and then generates products with a shorter molecule chain. However, it is noted (from Table 1) that not only the strength of the acidity varied across the Sbeta catalysts but also the mesoporous volume decreased significantly with the synthesis time of the catalysts. Combined with the results as shown in Figure 7A–H, it was inferred that a high mesoporosity correlated with cracked products with high carbon numbers, while a low mesoporosity to massive light gaseous products.^[^ ^10^, ^23^ ^]^ The higher the mesoporosity, the faster the diffusion rate (Figure 4), and then the less deep cracking, which offers the final products with a longer molecule chain.

#### 2.2.3 Polyethylene Hydrocracking Over In Situ NH_3_ Poisoning of the Zeolite Beta Catalysts

**Figure 8:**
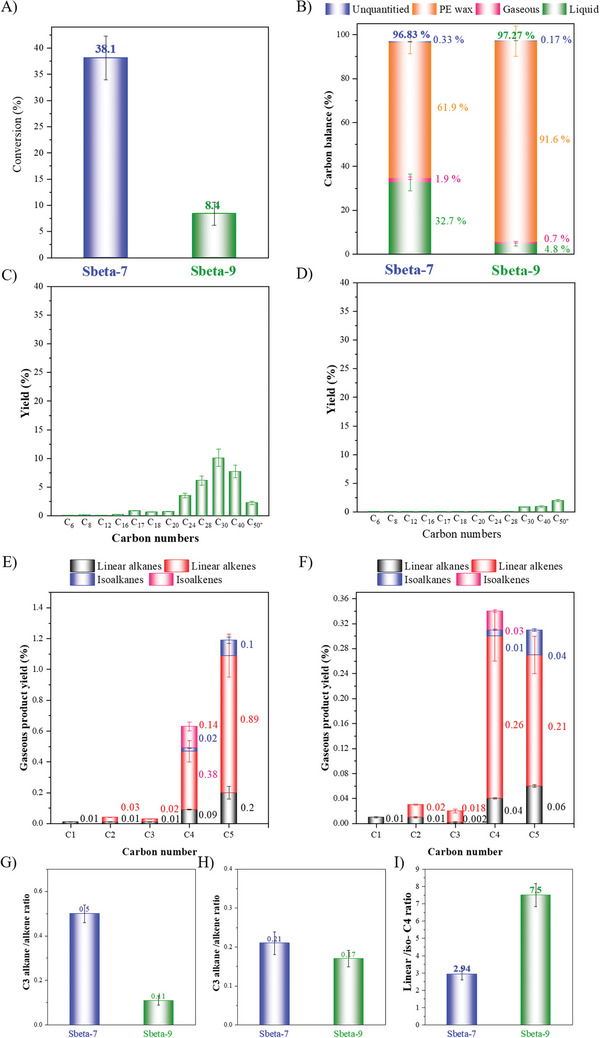
A) The conversions of PE cracking reaction over Sbeta‐7 and Sbeta‐9 after in situ NH_3_ poison. B) The corresponding products carbon balance. The liquid products distribution. C) Sbeta‐7. D) Sbeta‐9. Gaseous products distribution. E) Sbeta‐7. F) Sbeta‐9. G) Ratio of C_3_ alkanes/alkenes. H) Ratio of C_4_ alkanes/alkenes. I) Ratio of linear/*iso*‐C_4_. The number in the sample name stands for the time used for the synthesis of the zeolite beta catalysts. Reaction conditions: 200 mg of PE, 20 g of cyclohexane and 200 mg of poisoned catalyst; 2.0 MPa H_2_ atmosphere, 500 rpm, 260 °C, 240 min.

To further explore the effect of acidity on the hydrocracking of PE, Sbeta‐7 and Sbeta‐9 were studied by an in situ NH_3_ poisoning strategy proposed by our research group,^[^ ^55b^ ^]^ and then investigated in a H_2_ atmosphere (**Figure** **8**). After in situ NH_3_ poisoning, the conversion was significantly reduced, for Sbeta‐9, this value was reduced from 100% to 8.4%. This reduction was attributed to that reactant molecules could not reach the acid sites and, hence, blocked the micropores. The effect was smaller (100% vs 38.1%) on the Sbeta‐7 catalyst (see Table 1) and consistent that more of the PE could be converted by external acid sites on this catalyst. The carbon balances in Figure 8B show that liquid hydrocarbons appeared in the hydrocracking products of the poisoned Sbeta‐9 catalyst, while only a small amount of gas products formed, which was in stark contrast to the product distribution using non‐poisoned Sbeta‐9. The NH_3_ poisoning inhibited the deep hydrocracking of the products. In addition, the carbon number distributions of the cracking products after the NH_3_ poisoning (Figure 8C,D) were completely different from those using non‐poisoned catalysts (Figure 7C,D). After poisoning with NH_3_, the Sbeta‐7 and −9 catalysts delivered products within the range of lubricating base oils (C_24_ to C_50_^+^). These findings are consistent with that strong acid centers are needed to achieve deep cracking of PE to obtain small molecules, which is also consistent with the DFT analysis (Table S5, Supporting Information). For the small amount of gas produced with the poisoned Sbeta‐7 and Sbeta‐9 (Figure 8E,F), it was noted that mainly C_4_ and C_5_ olefins formed, and the hydrogen transfer reaction of lighter olefins was limited. The ratio of C_3_ alkanes/alkenes, C_4_ alkanes/alkenes, and linear/*iso*‐C_4_ in the gaseous products are presented in Figure 8G–I. When compared with the “HTIs” of products before the catalyst poisoning, both the C_3_ and C_4_ products using a NH_3_ poisoned catalyst are greatly reduced, which indicate that the hydrogen transfer reaction mainly occurred on the strong acidic sites, which is consistent with the literature.^[^ ^55b^ ^]^ In addition, the ratios of linear/*iso*‐C_4_ in the cracked products after poisoning are much higher, suggesting that it is difficult for the residual weak acid sites to isomerize linear‐C_4_.

**Figure 9:**
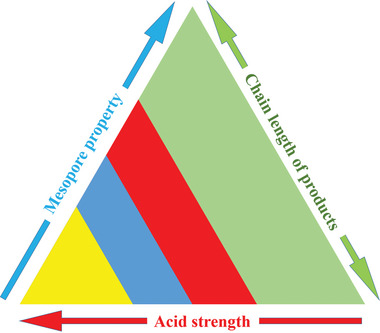
Triangle relationship of porosity‐acidity‐selectivity in the cracking or hydrocracking of polyethylene with zeolite beta catalysts of different character.

The above‐mentioned results suggest that the porosity and acidity of the Sbeta catalysts are of great importance for the catalytic cracking of PE. The structure‐function relationship between the porosity and acidity of the zeolite beta catalysts, and their catalytic performance for the catalytic cracking and hydrocracking of PE is illustrated in **Figure** **9**. The pure zeolite beta catalysts had high cracking and hydrocracking abilities. Such a highly crystalline zeolite beta has a small mesoporosity and a high acidity, and corresponding to a short chain‐length of the products. By controlling the crystallization time it is shown here that it is possible to tune both the mesoporosity as well as the acidity of the corresponding Sbeta zeolite. In turn, this means that by optimizing these properties of the catalyst, it is possible to control the selectivity of the products toward compositions relevant for different products such as gasoline, diesel oil, or lubricating base oil. Especially, it was noted that Sbeta‐3 with abundant mesopores and moderate acid strength enables that PE can be mainly cracked into lubricating base oil (88.7%). Furthermore, to assess the stability of the catalysts following the hydrocracking reaction, we regenerated the recovered catalyst at 550 °C and conducted three cyclic experiments. The subsequent analysis (Figure S11, Supporting Information) reveals negligible alterations in PE conversions and product selectivities relative to the initial cycle, underscoring the remarkable stability exhibited by the catalysts.

## 3 Conclusion

Zeolite beta catalysts with different degrees of micro‐ and mesoporosity, acidity and morphology were prepared via a solid conversion route using 2D ordered mesoporous SBA‐15 as a solid silicon source. A gradual collapse of the mesopores was accompanied by a formation of crystalline and microporous zeolite beta. Some of the X‐ray amorphous catalysts had a proto‐zeolite framework structure. The product composition of the catalytic cracking of PE was strongly affected by the mesoporous structure and acidity of the catalyst. With catalysts with low crystallinity and acidity, and high mesoporosity, a fast escape was promoted and a deep cracking of intermediate products was avoided, and a more lubricating base oil was obtained. Catalysts with high crystallinity, high acidity, and a low mesoporosity – well developed zeolite beta –caused multiple cracking of PE into hydrocarbons of mainly gaseous character. A triangular relationship of the porosity‐acidity‐selectivity during PE cracking was established. Via hydrocracking tests, it was shown that the pure zeolite catalyst could activate H_2_, and that the strong acid centers were the main active sites of hydrogen activation.

The main hypothesis in this study was that it is possible to tune the properties of cracking and hydrocracking of PE using various catalysts of the zeolite beta kind with different acidity and porosity. This extends on earlier studies of embryonic regular zeolite ZSM‐5 catalysts for the cracking of polypropylene by Tarach et al.,^[^ ^24^ ^]^ which did not involve the use of SBA‐15 in the synthesis. In the utilization of zeolite‐based catalysts for the treatment of macromolecular or supermolecular waste, pivotal considerations encompass the mitigation of diffusion constraints and the fine‐tuning of acid properties, both of which have profound implications on conversion efficiency and product selectivity. These factors hold significant relevance in optimizing the performance of such catalytic systems.

The catalytic cracking of PE on embryonic and well‐crystallized zeolite beta catalysts suggested that PE waste could be controllably cracked and hydrocracked into different valuable products by selecting catalysts with different porosity and acidity. The control of the cracking of the PE surpassed those observed by Caldeira et al. for the cracking of high‐density PE with a regular zeolite beta and a proto‐zeolite beta catalysts synthesized in the presence of hexadecyltrimethlyammonium bromide,^[^ ^56^ ^]^ but supported in general the findings of the control of the product distribution. The structure‐function relationships for catalysts of different crystallinity were revealed.

As a vision of future work, it is noted here that through a rational design of heterogeneous zeolite‐based solid acid catalysts, it may be possible to control the cracking of long‐chain polyolefin plastics waste into a large range of different valuable liquid products including gas‐diesel oil and lubricating base oil.

## 4 Experimental Section

### Chemical Reagents

Tetraethyl ammonium hydroxide solution (TEAOH, 25 wt% in water, Aladdin Reagent Co., Ltd.), tetraethyl ammonium bromide (TEABr, 98 wt%, Aladdin Reagent Co., Ltd.), sodium aluminate (Al_2_O_3_, ≥ 41%, Na_2_O ≥ 37%, Sinopharm Co., Ltd.), commercial SBA‐15 (Xianfeng Reagent Co., Ltd.), obsoleting PE wax (Mw = 2500‐3500, Fumei Co., Ltd.) and deionized water (H_2_O). All the materials are directly used without further purification.

### Synthesis of Embryonic Zeolite Beta

Zeolite beta was prepared with the following molar chemical composition (1589 H_2_O: 6 TEAOH: 18.7 TEABr: 1.5 Na_2_O: 1 Al_2_O_3_: 25 SiO_2_). In a typical synthesis, H_2_O (40 g), TEAOH (8 g), NaAlO_2_ (0.4 g), and TEABr (8 g) were added into a glass container, after the solids were completely dissolved, commercial SBA‐15 (2.4 g) was added into the above solution. After stirring vigorously at room temperature for 3 h, the mixtures were transferred to PP petri dishes, and placed in an oven at 50 °C to dry for 12 h. The dried solids were loaded into a polytetrafluoroethylene container and transferred to an autoclave, where 10 g of deionized water was added while keeping deionized water isolated from solid powders. The autoclaves were transferred into an oven at 140 °C for solid conversion, then, the as‐synthesized solid products were washed to neutral with deionized water, and dried in an electric blast drying oven at a set temperature of 100 °C for 12 h.

All samples were calcined in air at 550 °C for 6 h to remove the template, ion exchange was carried out with 1m NH_4_Cl at a solid‐liquid ratio of 1:30 at room temperature. The fresh solution was replaced every 2 h for three times. The protonic form samples were prepared by calcinating the NH^+^_4_ form samples for 6 h at 550 °C in air, and denoted as Sbeta‐t, “t” was the crystalline time (h).

### Characterization

Crystal structure analysis of the zeolite beta crystals was performed using X‐ray powder diffraction method, and the data was recorded on a Shimadzu XRD‐6000 diffractometer with Cu Kα radiation (λ = 0.15 406 nm), 40 kV, 30 mA. IR were recorded on a Shimadzu FT‐IR 8400 spectrometer using a transmission mode configuration and samples were dispersed in KBr pellets. IR spectra of the pyridine (Py) adsorbed sample were also recorded on the same IR spectrometer, where the samples were activated under near‐vacuum conditions of 10 mPa at 400 °C for 4 h. After cooling to 150 °C, Py was exposed to the sample for 30 min, then spectra with adsorbed Py were recorded at temperatures of 150, 250, and 350 °C. UV‐Raman spectra were recorded on a Horiba Jobin‐Yvon LabRAM HR800 spectrometer. Crystal morphologies and sizes were investigated by SEM using a Hitachi S‐4800 microscope equipped with an energy‐dispersive X‐ray spectroscopy device (EDS) and by TEM using a JEOL JEM 2100 microscope. Solid‐state MAS NMR spectra of ^29^Si and ^27^Al were carried on Bruker Avance III 600 spectrometer, and no additional activation steps were required before sample analysis. ^29^Si single pulse experiments were recorded using π/6 excitation pulses, 2048 transients, and 20 s recycle time at 99.35 MHz. Zero calibration was carried out using tetramethylsilane (TMS) as the standard; ^27^Al MAS NMR spectra were recorded at 104.26 MHz with single pulse π/12 excitation and the corresponding recycle delay was 0.5 s, a sample spinning rate of 8 kHz using 4 mm rotors. Zero calibration was carried out using 1 M aluminum nitrate (Al(NO_3_)_3_) solution as the standard. A NOVA 1200e N_2_ adsorption‐desorption gas adsorption analyzer was used at 77 K. A nonlocalized (NL)‐DFT model was used to analyze the adsorption branch of the N_2_ adsorption isotherm to derive an estimate of the PSD. The microporous surface areas (S_mic_) were calculated through the *t*‐plot method. The acid properties of as‐prepared samples were investigated by NH_3_–Temperature Programmed Desorption (TPD) by a TP‐5076 TPD/Temperature Programmed Reduction (TPR) apparatus. Typically, 100 mg of a sample was heated at 550 °C for 1 h in a flow rate of 30 mL min^−1^ in a helium (He) atmosphere to remove adsorbed impurities. After cooling to 120 °C, the samples were exposed to NH_3_/N_2_ mixture (5%) at a flow rate of 30 mL min^−1^ for 0.5 h. Then, physically absorbed NH_3_ was removed under pure He flow of 30 mL min^−1^ for 1 h. Subsequently, samples with adsorbed NH_3_ were subjected to a He flow of 30 mL min^−1^ at increasingly higher temperatures, from 120 to 700 °C with a heating rate of 10 °C min^−1^. The desorbed NH_3_ was accordingly recorded by a thermal conductivity detector (TCD). TG‐DSC analysis was used to explore the coking nature and amount of used catalysts. This analysis was performed on a Netzch SAT 449F3 instrument with a temperature ramping rate of 10 °C min^−1^ from 100 to 800 °C under an airflow. The diffusivity of toluene on embryonic and well‐crystalline beta zeolites was determined by the ZLC method. The procedures were as follows: 1 to 2 mg of samples were loaded into a cuvette consisting of two porous metal sieve plates and activated at a temperature of 280 °C for 12 h under a flow of 30 mL min^−1^ of high‐purity He as a carrier gas to remove adsorbed impurities. Then the sample was cooled down to the adsorption temperature at which toluene in He (≈1000 ppm) was subjected to the sample at a flow rate of 80 mL min^−1^ for 1 h. At the end of the adsorption process, the gas flow was quickly switched to a high‐purity He gas at a flow rate of 80 mL min^−1^, and the desorbing toluene was recorded with a flame ionization detector (FID). The data points for the toluene desorption, over the full time range, were subjected to a regression analysis using a Matlab code to obtain the *D*_eff_/*R* ^2^ and *L* parameters. More detailed information was presented in the supplementary information.

### Catalytic Tests

*Hydrocracking of Polyethylene*: Hydrocracking of PE was studied in a 50 mL stainless steel autoclave. Before the reaction, the H‐type zeolite beta catalysts were activated at a temperature of 550 °C for 4 h to remove impurity gas and water molecules adsorbed on the zeolite surfaces. Typically, 200 mg of PE, 20 g of cyclohexane as a solvent, and 200 mg of an activated catalyst were mixed in the autoclaves. The air inside the autoclave was substituted by H_2_, and the process was repeated four times, with subsequent exposure of H_2_ at a pressure of 2.0 MPa. Then the catalytic system was heated up from a temperature of 20 °C at a rate of 5 °C min^−1^ to 260 °C. The reaction systems were conducted at 260 °C for 240 min under a stirring speed of 500 rpm. After the reaction, the autoclave was cooled in air. The gaseous products were collected in a gas bag and analyzed by gas chromatography (Agilent 7820A) equipped with an FID detector and an HP‐AL/KCL+PT chromatographic column (Agilent Co., Ltd.). Then, a centrifuge was applied to separate the catalyst and unreacted PE from the liquid. A gas chromatography (Agilent 7890B) equipped with an FID detector and a stainless steel chromatographic column (Sinopec Research Institute of Petroleum Processing Co., Ltd.) was employed to analyze the cracking products. More detailed information could be viewed in the supporting information.

*Catalytic Cracking of Polyethylene*: The catalytic cracking process of PE was similar to the hydrocracking process described above, with the only difference being that high‐purity N_2_ was used instead of H_2_.

*PE Hydrocracking Over* In Situ *NH_3_ Poisoning of the Zeolite Beta Catalysts*: The H‐type of the zeolite beta catalyst was activated at 550 °C for 4 h, cooled down to room temperature, and then processed using a 5% NH_3_/N_2_ mixture at a flow rate of 50 mL min^−1^ for 30 min to obtain the in situ poisoned catalysts. The other procedures of the reaction were consistent with the PE hydrocracking process, but the catalysts used were the in situ poisoned catalysts described above.

### Calculation Details

All calculations were conducted with Materials Studio.^[^ ^57^ ^]^ software (Dmol3 module). The electron‐ion interactions were characterized using the PAW (projector augmented wave) method with a truncation energy of 500 eV.^[^ ^58^ ^]^ The generalized gradient approximation (GGA) method and Becke‐Perdew‐Wang (BP).^[^ ^59^ ^]^ exchange‐correlation generalized functions were used for the optimization of structure and the calculation of energy. More detailed information could be viewed in the SI.

## Conflict of Interest

The authors declare no conflict of interest.

## Supporting information

Supporting Information

## Data Availability

The data that support the findings of this study are available from the corresponding author upon reasonable request.
