# Supplementary material for: Selective and Controllable Cracking of Polyethylene Waste by Beta Zeolites with Different Mesoporosity and Crystallinity
Source: Adv Sci (Weinh). 2024 Jul 8;11(34):2404426. doi: 10.1002/advs.202404426 (PMC11425912; doi:10.1002/advs.202404426)
Supplement: Supplementary file 1 — Supporting Information [file ADVS-11-2404426-s001.pdf]

ADVANCED SCIENCE
Open Access

Supporting Information

for *Adv. Sci.*, DOI 10.1002/advs.202404426

Selective and Controllable Cracking of Polyethylene Waste by Beta Zeolites with Different Mesoporosity and Crystallinity

Yanchao Liu, Weijiong Dai, Jiajun Zheng, Yanze Du, Quanhua Wang, Niklas Hedin*, Bo Qin* and Ruifeng Li**

Supporting Information

Selective and controllable cracking of polyethylene waste by beta zeolites with different mesoporosity and crystallinity

Yanchao Liu, Weijiong Dai, Jiajun Zheng, Yanze Du, Quanhua Wang, Niklas Hedin*, Bo Qin*, Ruifeng Li**

1. Analysis of gaseous products

The gas chromatogram was calibrated using a configured standard gas (CH_4: 0.97%, C_2H_4: 0.632%, C_2H_6: 0.271%, C_3H_6: 2.99%, C_3H_8: 0.371%, *n*-C_4H_8: 0.749%, *iso*-C_4H_8: 0.795%, *n*-C_4H_{10}: 0.145%, *iso*-C_4H_{10}: 0.470%, *n*-C_5H_{10}: 0.070%, *iso*-C_5H_{10}: 0.244%, *n*-C_5H_{12}: 0.494%) and the absolute content of the gaseous products was analyzed. The gaseous products were injected through a gas bag into a 6-way valve with a quantification ring, and the absolute content of each substance in the gaseous products was quantified based on the FID signal.

2. Analysis of liquid products

Retention times and relative contents of hydrocarbons were determined using standard solutions of C_6-C_{50} (Sinopec Research Institute of Petroleum Processing Co., Ltd.), and the absolute contents of various hydrocarbons in the liquids were quantified using mesitylene as an external standard.

To test for possible errors in the analysis of the C_5 and C_6 products, 1 g *n*-C_5 or *n*-C_6, respectively, were added to 20 g cyclohexane, heated under identical reaction conditions for 4 h and then cooled to room temperature. The recovered products were analyzed by gas chromatography and compared to the pre-reaction mixtures of *n*-C_5 (*n*-pentane) or *n*-C_6 (*n*-hexane), which demonstrated that only 13.8% of *n*-C_5 and 22.1% of *n*-C_6 products were cooled to the liquid phase after the reaction.

3. Calculation of carbon balance

The carbon balance results were obtained by calculating the absolute content of hydrocarbons contained in gaseous and liquid products, but both had a significant impact on carbon balance results due to the fact that the solvent shielded some of C_6 products, and also due to the fact that some of C_5 was not completely cooled and volatilized during

collection of liquid products. The unquantified fraction originated from components that could not be identified in the gas chromatograms, but which were reaction products.

4. Theoretical model of ZLC diffusion characterization

The diffusion of adsorbents in zeolite could be described by the model proposed by Crank. The calculation formulas were as follows.

$$\frac{C}{C_0}=2L\sum_{n=1}^{\infty}\frac{\exp\left(-\frac{\beta_n^2 D_{eff}t}{R^2}\right)}{[\beta_n^2+L(L-1)]} \quad (1)$$

$$\beta_n \cot\beta_n+L-1=0 \quad (2)$$

$$L=\frac{1}{3}\frac{FR^2}{KV_S D_{eff}} \quad (3)$$

In the above formula, C_0 was the initial concentration of the adsorbate (toluene); C was the concentration of adsorbate in the gas phase during desorption; β and L were the introduced parameters; F was the carrier gas flow rate; K was the Henry constant with a dimension of 1; V_s was the volume of adsorbent; D_{eff}/R^2 was the effective diffusion time constant; t was the time.

5. Calculation details

The Brillouin zone integrals were performed using a Monkhorst-Pack grid, and the structural optimization was performed by setting the force convergence criterion to 0.01 eV/Å, and the electron self-consistency convergence criterion to 10^{-4} eV. The LST-QST method was used to determine the transition states of the reaction. The optimized structures were then used to determine the transition states of reaction, and reaction paths between reactants and products were probed using frequency analysis. The optimized transition states were then further optimized using the proposed Newtonian method and each transition state was analytically verified using frequency analysis, and the harmonic frequencies were performed on the fully optimized structures with the displacements near the atomic equilibrium position set to ± 0.02 Å. For the optimized structures with imaginary frequencies, the linear minimization method proposed by Bučko et al. and Gutierrez-Acebo et al. The number of imaginary frequencies was reduced and the process was repeated until a transition state with only one imaginary frequency or an energy minimum without imaginary frequencies was found.

Table S1. Acid properties of Sbeta-3

	B acid sites[a)] [μmol g^{-1}]	L acid sites[a)] [μmol g^{-1}]	B/L
150 °C	62	26	2.4
250 °C	46	24	2.0
350 °C	33	22	1.5

a)B acid sites and L acid sites represent the Brønsted acid sites and Lewis acid sites, respectively.

C (pyridine on B acid sites) = 1.88IA(B)R^2 /W;

C (pyridine on L acid sites) = 1.42IA(L)R^2 /W.[1]

Table S2. The relative proportion of Si species under different coordination environment in the as-prepared catalysts.

Samples a)	Q^4 [%]	Q^3 [%]	Q^2 [%]
Sbeta-1	48.8	26.7	24.5
Sbeta-3	44.0	36.9	16.4
Sbeta-7	41.5	46.7	11.9

a)The number denotes the time of synthesis.

Table S3. The relative proportion of Al about hexa-coordinated (Al^{VI}), penta-coordinated (Al^{V}) and tetra-coordinated(Al^{IV}) species in the as-prepared catalysts.

Samples a)	Peak (δ=0ppm) Al^{VI} [%]	Peak (δ=30ppm) Al^{V} [%]	Peak (δ=52ppm) Al^{IV} [%]
Sbeta-1	26.7	41.6	31.7
Sbeta-3	17.5	46.4	36.1
Sbeta-7	15.8	39.7	44.5

a)The number denotes the time of synthesis.

Table S4. The acid amounts, effective acid density and crystal size of the catalysts.

Samples a)	Sbeta-1	Sbeta-3	Sbeta-5	Sbeta-7	Sbeta-9	Sbeta-11	Sbeta-13
Acid density b) [mmol g^{-1}]	0.206	0.395	0.402	0.418	0.695	0.868	0.956
Effective acidity c) [mmol g^{-1}]	0.197	0.307	0.238	0.231	0.114	0.132	0.102
Crystal size d) [nm]	--	--	--	--	22	25	23

a)The number denotes the time of synthesis.

b)Decided by NH_3-TPD.

c)Determined by the equation as following: Effective acid density = Acid density× S_{ext} / S_{BET}.[2]

d)Determined by Scherrer equation.

Table S5. Cracking reaction energy barriers for hydrocarbons with different carbon numbers.

Reactants	Products	Energy of barrier [kJ mol^{-1}]
C_3	$C_1 + C_2^{=}$	561

C_5	$C_2 + C_3^=$	487
C_{10}	$C_5 + C_5^=$	450
	$C_7 + C_3^=$	466
C_{20}	$C_{10} + C_{10}^=$	390
	$C_{17} + C_3^=$	441
C_{40}	$C_{20} + C_{20}^=$	339
	$C_{37} + C_3^=$	419
C_{80}	$C_{40} + C_{40}^=$	285
	$C_{77} + C_3^=$	396

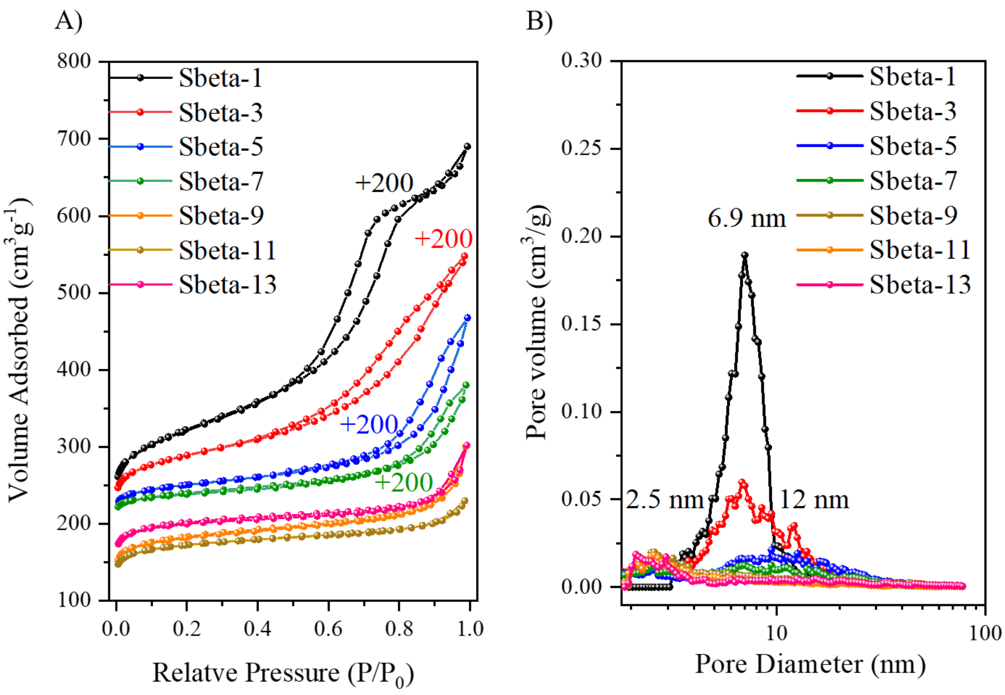

Figure S1. A) N_2 adsorption-desorption isotherms. B) The corresponding DFT pore size distribution curves of embryonic and well-crystallized beta zeolite samples. The number in the sample name stands for the time used for the synthesis of the zeolite beta catalysts.

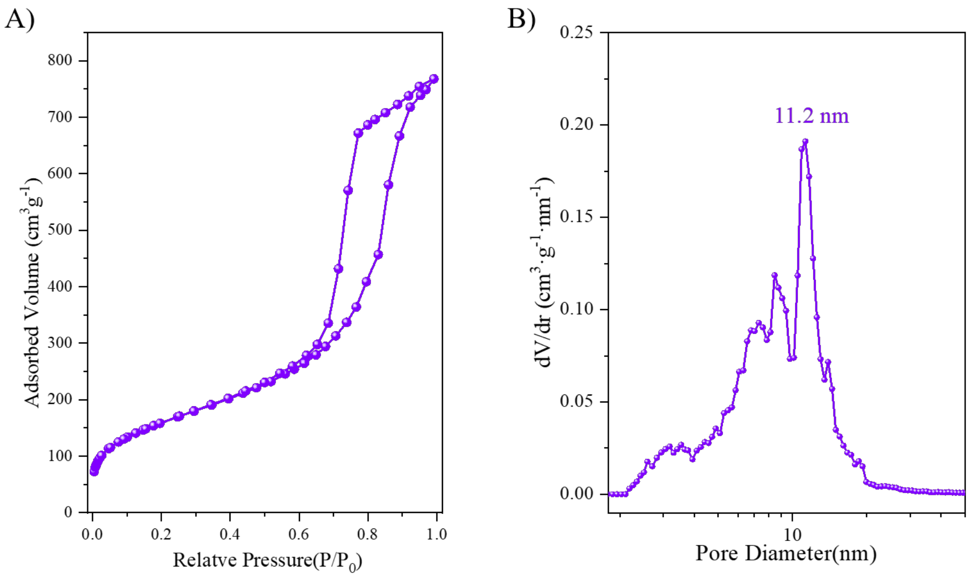

Figure S2. A) N_2 adsorption-desorption isotherms. B) The corresponding DFT pore size distribution of Si source SBA-15.

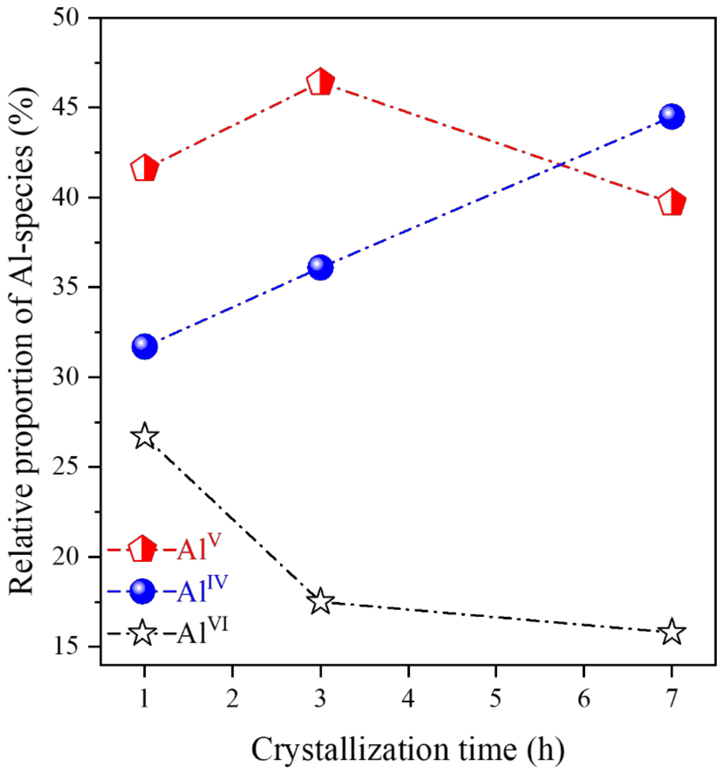

Figure S3. The effect of crystallization time on the relative proportion of the different coordinated Al-species.

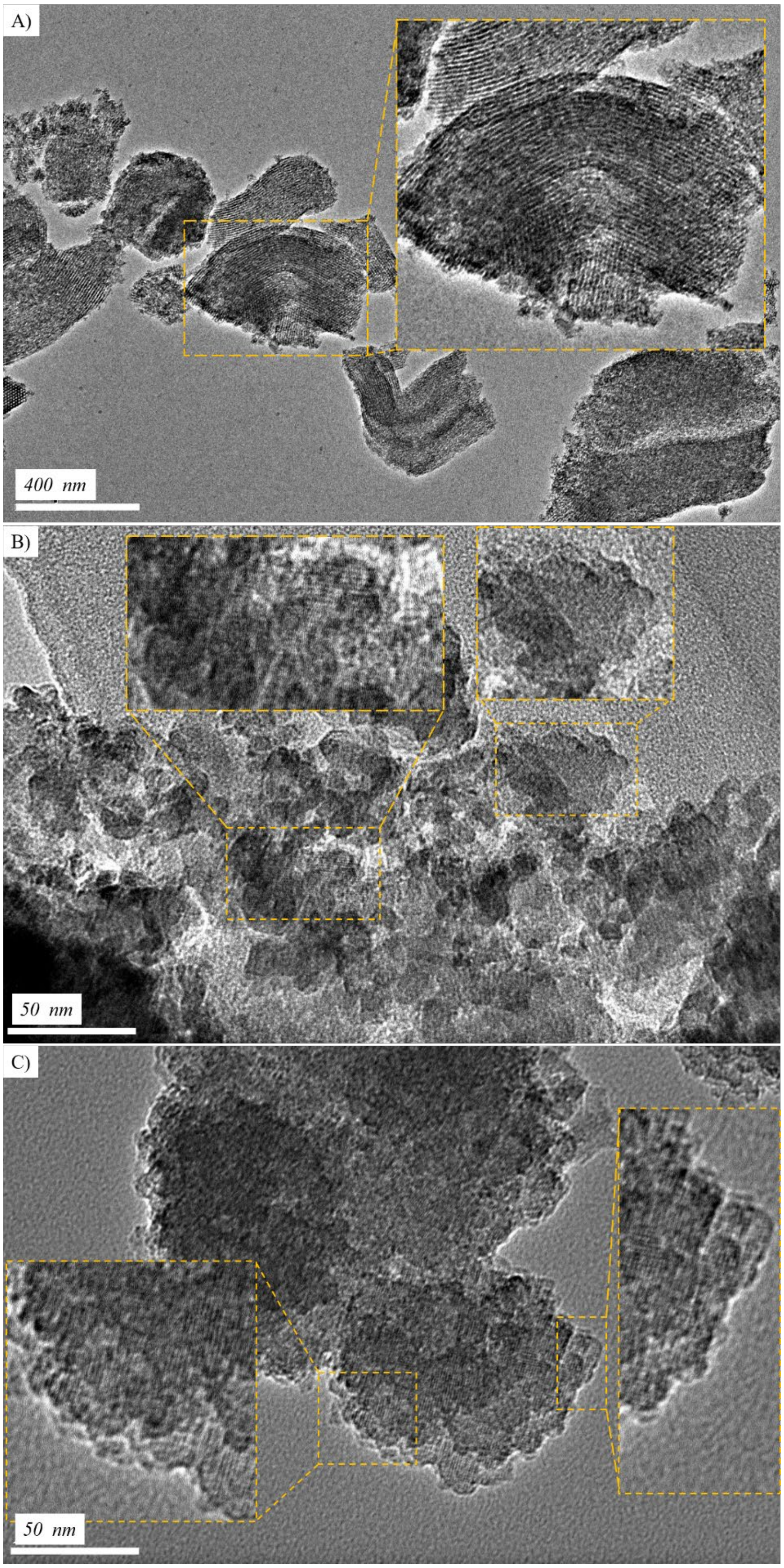

Figure S4. TEM images of the pristine SBA-15 and the as-synthesized samples. A) SBA-15. B) Sbeta-7. C) Sbeta-9. The number in the sample name stands for the time used for the synthesis of the zeolite beta catalysts.

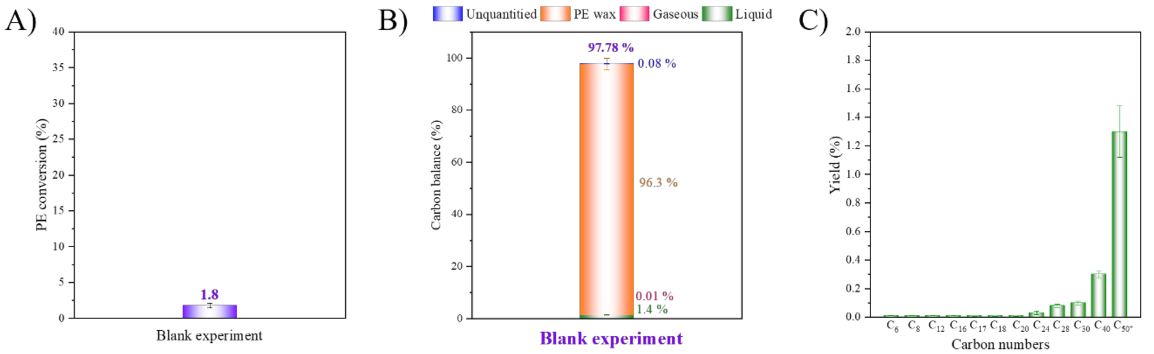

Figure S5. A) The conversion of PE thermal cracking reaction without catalyst. B) The corresponding products carbon balance. C) The liquid products yield. Reaction conditions: 200 mg of PE, 20 g of cyclohexane; 2.0 MPa N_2 atmosphere, 500 rpm, 260 °C, 240 min.

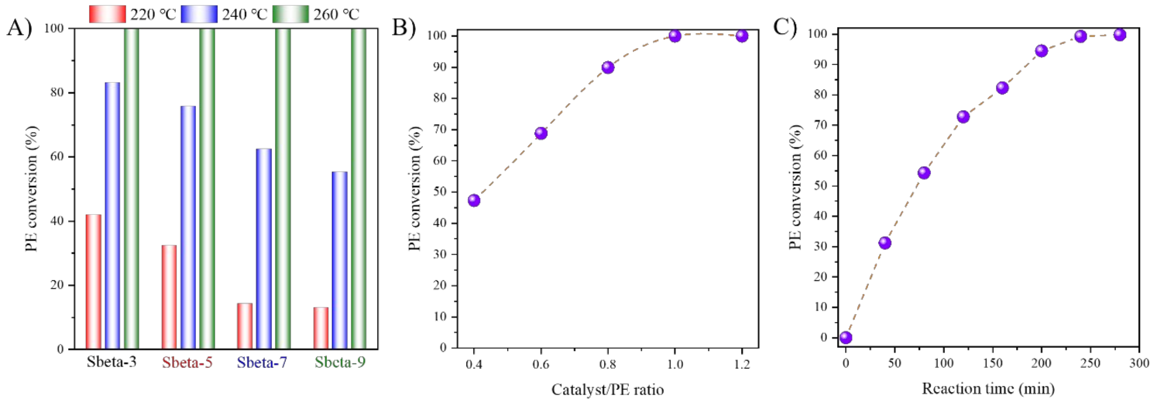

Figure S6. A) Conversion of PE over Sbeta-t catalysts at different reaction temperatures. (Reaction conditions: 200 mg of PE, 20 g of cyclohexane, 200 mg of catalyst; 2.0 MPa N_2 atmosphere, 500 rpm, 240 min). B) Ratio of catalyst/PE versus PE conversion on Sbeta-9 catalysts. (Reaction conditions: 200 mg of PE, 20 g of cyclohexane; 2.0 MPa N_2 atmosphere, 500 rpm, 260 °C, 240 min). C) Reaction time versus PE conversion on Sbeta-9 catalysts. (Reaction conditions: 200 mg of PE, 20 g of cyclohexane, 200 mg of catalyst; 2.0 MPa N_2 atmosphere, 500 rpm, 260 °C).

Reaction temperature: The cracking reaction of PE is a highly endothermic process, and then the higher reaction temperature, the higher conversion of PE. As shown in **Figure S6A**, the elevated temperature is in favour of the catalytic cracking of PE. However, the excessive reaction temperature for example 300 °C also promotes the secondary reactions, this reduces the yield of liquid fractions while increase the yield of gaseous small molecules in the final products.

Catalyst/PE ratio**:** As shown in **Figure 6SB**, the catalyst/PE ratio also strongly affects the conversion of PE on Sbeta-9 catalyst. Obviously, the increased catalyst/PE ratio means relatively increased acid sites.

Reaction time: Due to the large kinetic diameter of PE molecules, PE diffuses and approaches the acid centers at a considerably slower rate compared to smaller molecular reactants, resulting in a lower turnover frequency of the acid centers. To enhance PE conversion, extending the reaction time are very effective. As displayed in **Figure S6C**, the conversion of PE is positive correlation with prolonged reaction time. After 240 min, PE can be fully converted under the condition of: 200 mg of PE, 20 g of cyclohexane, 200 mg of catalyst; 2.0 MPa N_2 atmosphere, 500 rpm, 260 °C.

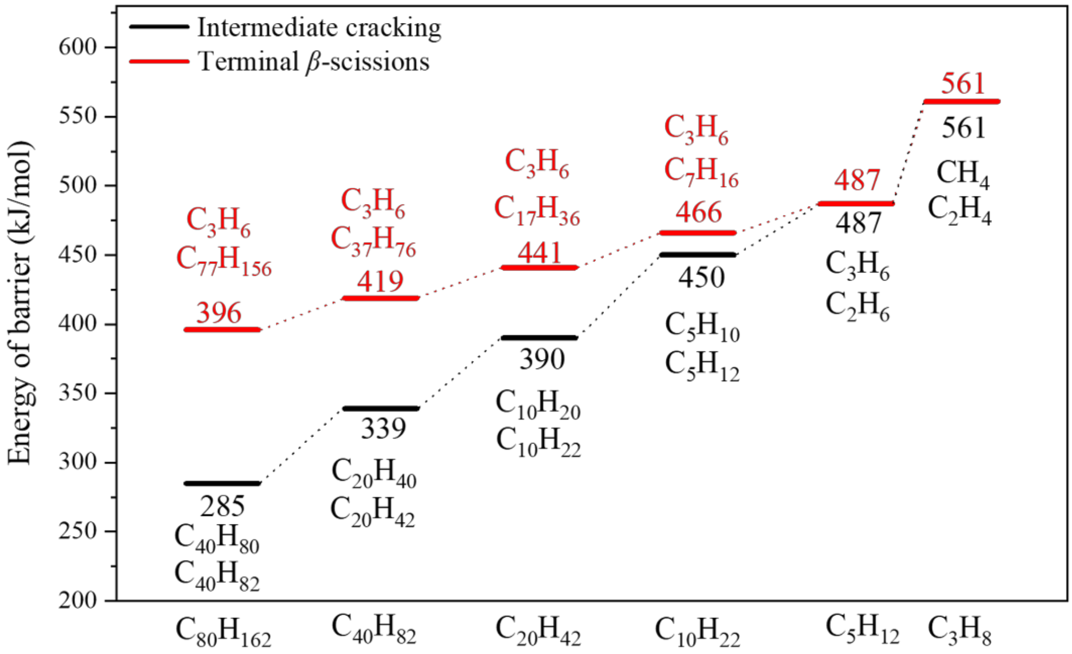

Figure S7. Calculation of reaction energy barriers for catalytic cracking of hydrocarbons with different carbon numbers through two kinds cracking model.

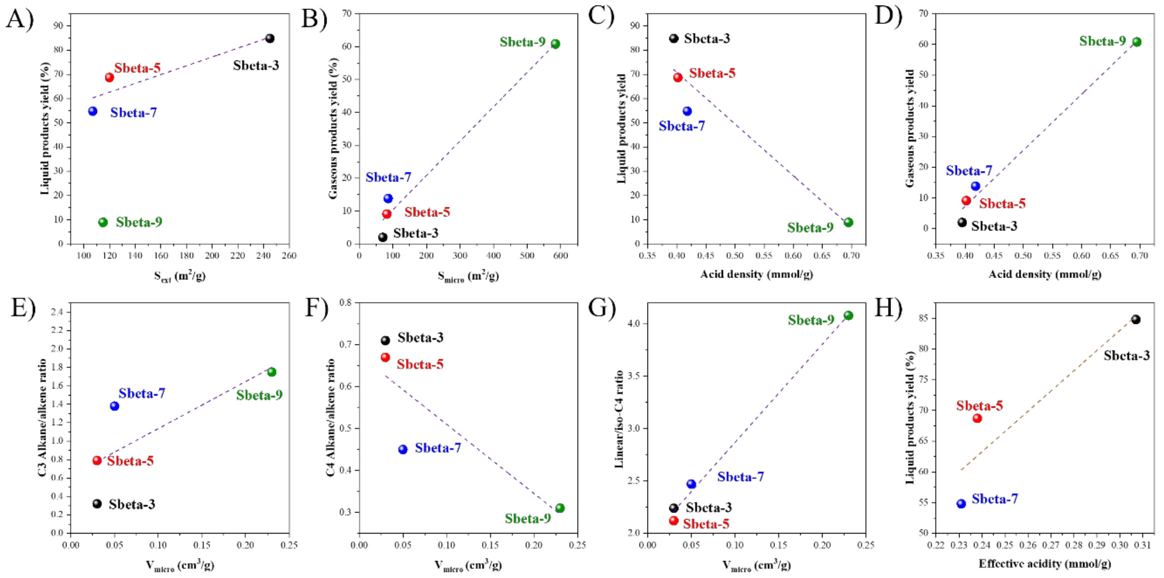

Figure S8. A) Catalyst mesoporous surface area versus liquid product yields. B) Catalyst microporous surface area versus gaseous product yields. C) Catalyst acid density versus liquid product yields. D) Catalyst acid density versus gaseous product yields. E) Catalyst microporous volume versus C_3 alkane/alkene ratios. F) Catalyst microporous volume versus C_4 alkane/alkene ratios. G) Catalyst microporous volume versus linear/*iso*-C_4 ratios. H) Effective acid density versus liquid product yields of the catalysts in the cracking of PE. Reaction conditions: 200 mg of PE, 20 g of cyclohexane and 200 mg of catalyst; 2.0 MPa N_2 atmosphere, 500 rpm, 260 °C, 240 min.

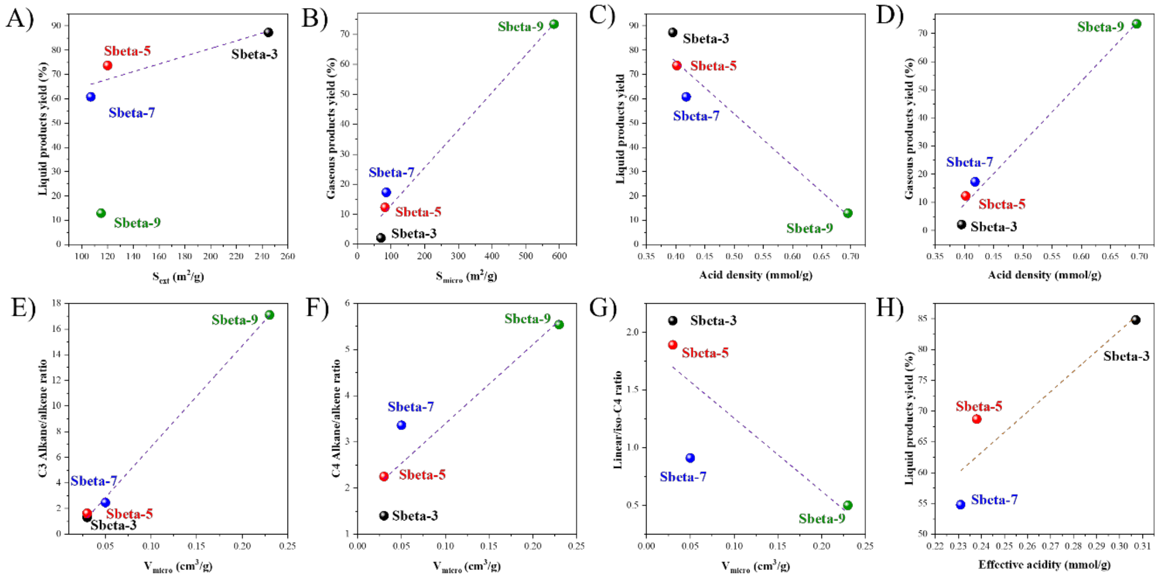

Figure S9. A) Catalyst mesoporous surface area versus liquid product yields. B) Catalyst microporous surface area versus gaseous product yields. C) Catalyst acid density versus liquid product yields. D) Catalyst acid density versus gaseous product yields. E) Catalyst microporous volume versus C_3 alkane/alkene ratios. F) Catalyst microporous volume versus C_4 alkane/alkene ratios. G) Catalyst microporous volume versus linear/*iso*-C_4 ratios. H) Effective acid density versus liquid product yields of the catalysts in the hydrocracking of PE.

Reaction conditions: 200 mg of PE, 20 g of cyclohexane and 200 mg of catalyst; 2.0 MPa H_2 atmosphere, 500 rpm, 260 °C, 240 min.

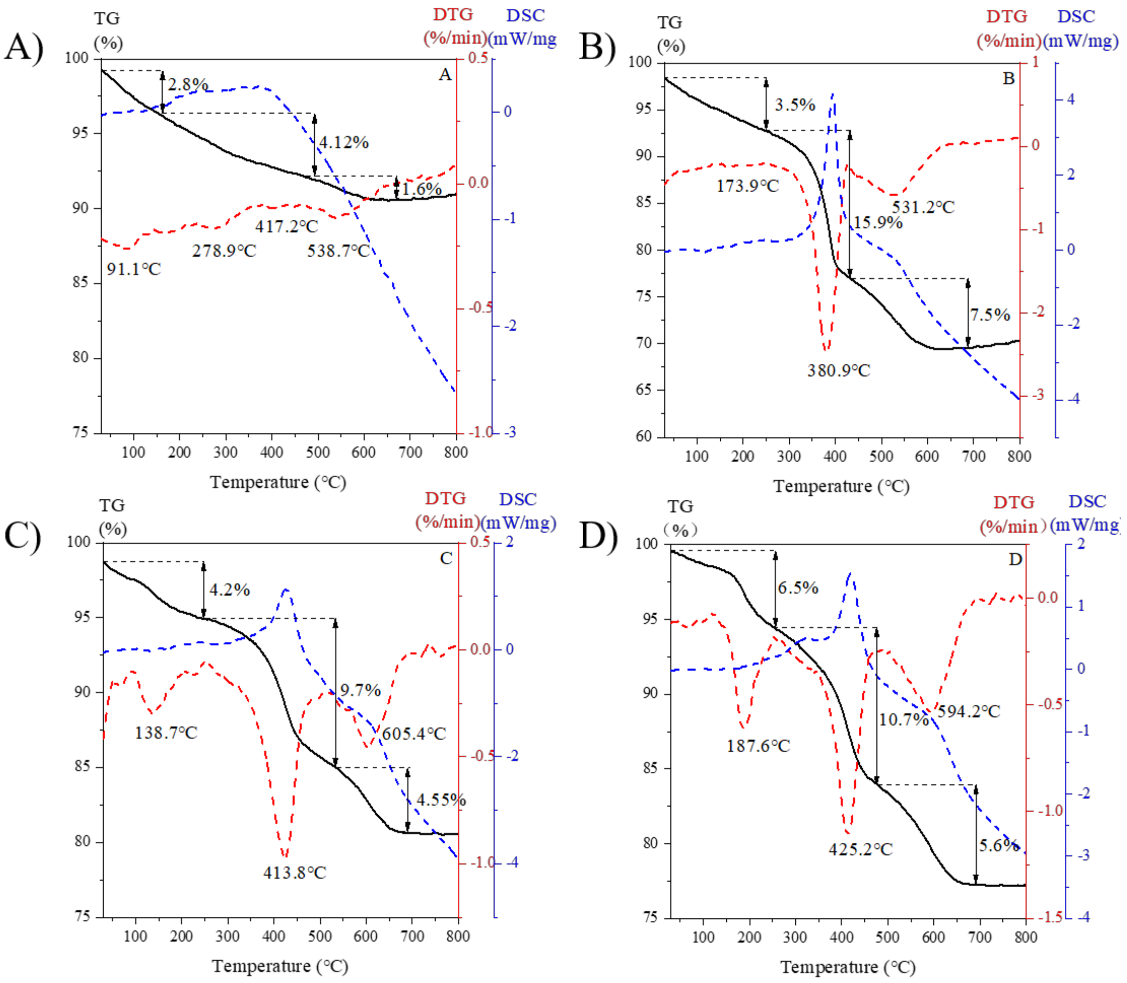

Figure S10. TG-DSC curves of the spent catalysts after tested in catalytic cracking of PE. A) Sbeta-3. B) Sbeta-5. C) Sbeta-7. D) Sbeta-9. The number in the sample name stands for the time used for the synthesis of the zeolite beta catalysts.

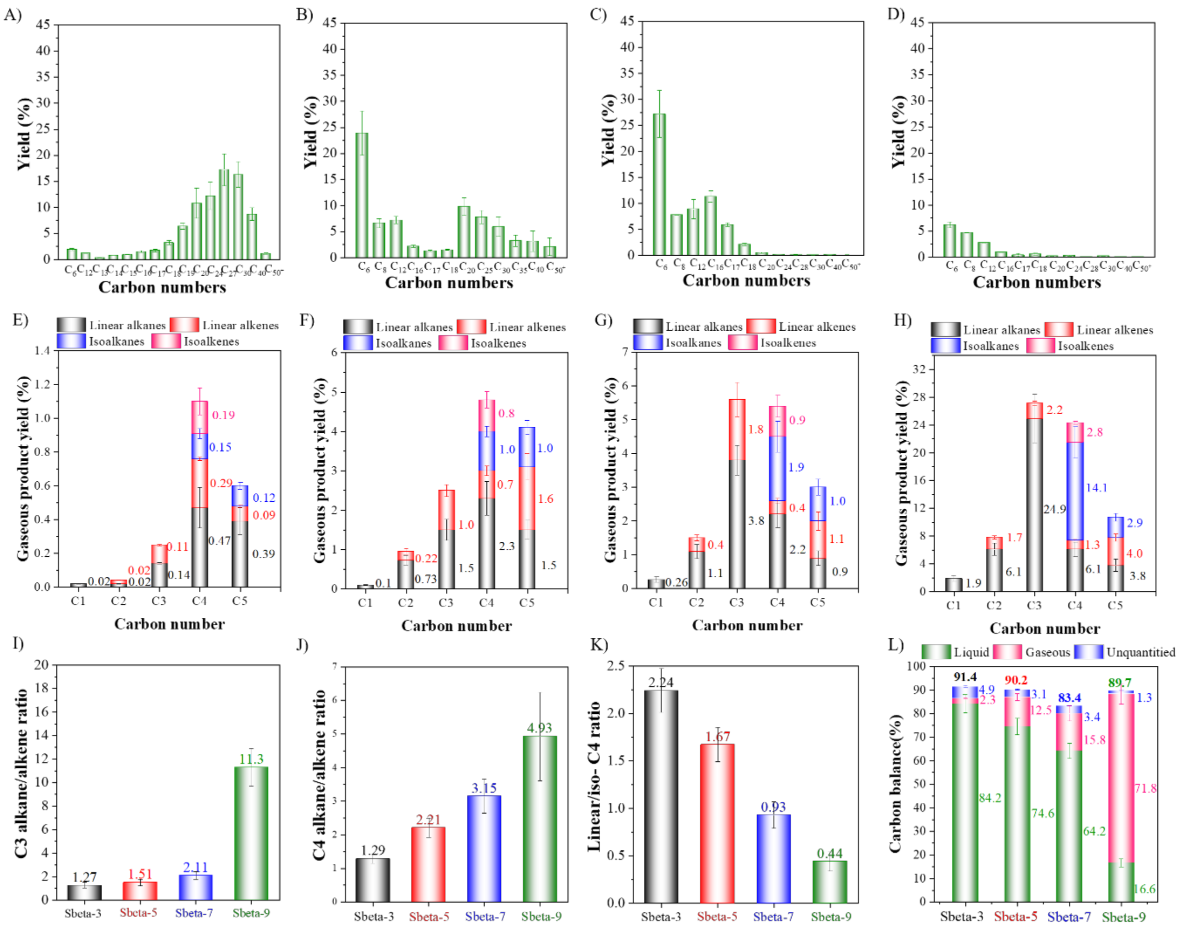

Figure S11. The liquid products distribution. A) Sbeta-3. B) Sbeta-5. C) Sbeta-7. D) Sbeta-9. Corresponding gaseous products distribution. E) Sbeta-3. F) Sbeta-5. G) Sbeta-7. H) Sbeta-9. I) The ratio of C_3 alkanes/alkenes. J) The ratio of C_4 alkanes/alkenes. K) The ratio of linear/*iso*-C_4 in gaseous products. L) The product's carbon balance over four catalysts. The number in the sample name stands for the time used for the synthesis of the zeolite beta catalysts. The catalysts used were recovered catalysts from the PE hydrocracking reaction, which were then regenerated at 550 °C with an air atmosphere for 6 hours. Reaction conditions: 200 mg of PE, 20 g of cyclohexane and 200 mg of regenerated catalyst; 2.0 MPa H_2 atmosphere, 500 rpm, 260 °C, 240 min.

Reference

[1] C. A. Emeis, *J. Catal.* **1993**, *141*, 347.

[2] a) Y. Du, Q. Kong, Z. Gao, Z. Wang, J. Zheng, B. Qin, M. Pan, W. Li, R. Li, *Ind. Eng. Chem. Res.* **2018**, *57*, 7395; b) Q. Wang, L. Zhang, Z. Yao, Y. Guo, Z. Gao, J. Zheng, W. Li, B. Fan, Y. Wang, S. Chen, *Mater. Chem. Phys.* **2020**, *243*, 122610.
